# The Regenerating Adult Zebrafish Retina Recapitulates Developmental Fate Specification Programs

**DOI:** 10.3389/fcell.2020.617923

**Published:** 2021-02-01

**Authors:** Manuela Lahne, Margaret Brecker, Stuart E. Jones, David R. Hyde

**Affiliations:** ^1^Department of Biological Sciences, University of Notre Dame, Notre Dame, IN, United States; ^2^Center for Stem Cells and Regenerative Medicine, University of Notre Dame, Notre Dame, IN, United States; ^3^Center for Zebrafish Research, University of Notre Dame, Notre Dame, IN, United States

**Keywords:** Müller glia, retinal regeneration, competence factors, birth order, differentiation, zebrafish (*Brachydanio rerio*), neuronal progenitor cell

## Abstract

Adult zebrafish possess the remarkable capacity to regenerate neurons. In the damaged zebrafish retina, Müller glia reprogram and divide to produce neuronal progenitor cells (NPCs) that proliferate and differentiate into both lost neuronal cell types and those unaffected by the damage stimulus, which suggests that developmental specification/differentiation programs might be recapitulated during regeneration. Quantitative real-time polymerase chain reaction revealed that developmental competence factors are expressed following photoreceptor damage induced by intense light or in a genetic rod photoreceptor cell ablation model. In both light- and N-Methyl-D-aspartic acid (NMDA)-damaged adult zebrafish retinas, NPCs, but not proliferating Müller glia, expressed fluorescent reporters controlled by promoters of ganglion (*atoh7*), amacrine (*ptf1a*), bipolar (*vsx1*), or red cone photoreceptor cell competence factors (*thrb*) in a temporal expression sequence. In both damage paradigms, *atoh7:GFP* was expressed first, followed by *ptf1a:EGFP* and lastly, *vsx1:GFP*, whereas *thrb:Tomato* was observed in NPCs at the same time as *ptf1a:GFP* following light damage but shifted alongside *vsx1:GFP* in the NMDA-damaged retina. Moreover, HuC/D, indicative of ganglion and amacrine cell differentiation, colocalized with *atoh7:GFP* prior to *ptf1a:GFP* expression in the ganglion cell layer, which was followed by Zpr-1 expression (red/green cone photoreceptors) in *thrb:Tomato*-positive cells in the outer nuclear layer in both damage paradigms, mimicking the developmental differentiation sequence. However, comparing NMDA- to light-damaged retinas, the fraction of PCNA-positive cells expressing *atoh7:GFP* increased, that of *thrb:Tomato* and *vsx1:GFP* decreased, and that of *ptf1a:GFP* remained similar. To summarize, developmental cell specification programs were recapitulated during retinal regeneration, which adapted to account for the cell type lost.

## Introduction

**FIGURE 1:**
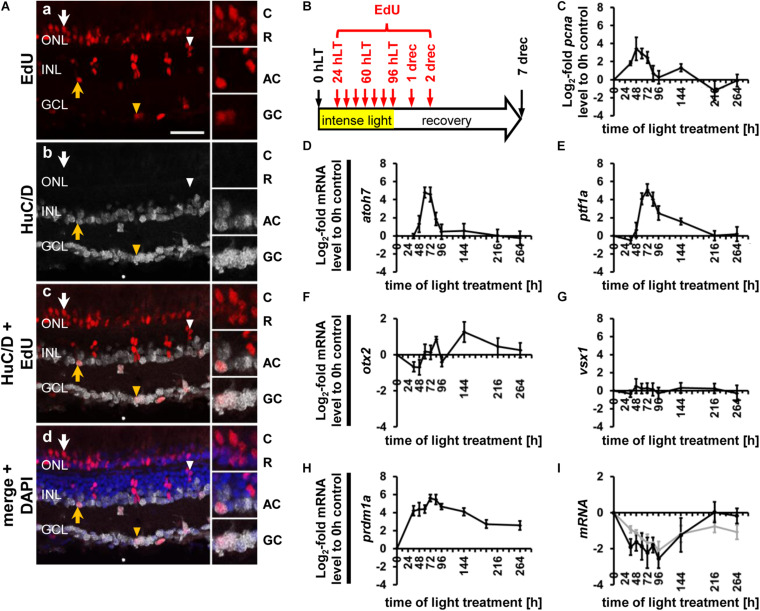
Generation of all neuronal cell types and expression of cell type specific developmental competence factors in the light-damaged retina. **(Aa–d)** Single z-plane confocal images of retinal sections from light-damaged EdU-injected *albino* zebrafish **(Aa,c,d)** at 7 days of recovery (drec) immunolabeled for the amacrine/ganglion cell marker, HuC/D **(Ab–d)**, and counterstained with the nuclear dye, DAPI **(Ad)**. Yellow arrowhead, GCL EdU-positive ganglion or amacrine cell; yellow arrow, INL EdU-positive amacrine cell; white arrowhead, EdU-positive and HuC/D-negative cell in the apical INL, the region where bipolar cells reside; white arrow, EdU-positive cell in the cone nuclear layer. Insets represent the EdU-positive cells in panels **(Aa–d)** (arrows; yellow arrowhead) displayed at higher magnification. Scale bar, 20 μm in panel **(Aa)**. **(B)** Schematic of the experimental paradigm: *albino* zebrafish were exposed to constant intense light for 96 h and subsequently recovered under normal light conditions until 7 drec. Fish were intraperitoneally injected with EdU at 24, 36, 48, 60, 72, 84, 96 hLT and at 1 and 2 drec (red arrows). AC, amacrine cell; C, cone photoreceptor cell; GC, ganglion cell; GCL, ganglion cell layer; hLT, hours of light treatment; INL, inner nuclear layer; ONL, outer nuclear layer; R, rod photoreceptor cell. **(C–I)** Line plots displaying mRNA expression levels as log_2_-fold changes relative to 0 h controls for proliferation marker, *pcna* **(C)** and genes required for the developmental specification of retinal neurons **(D–I)** during light damage-induced retinal regeneration (0, 36, 48, 60, 72, 84, 96 hLT, 2, 5, 7 drec): **(D)** *atoh7* (ganglion cells); **(E)** *ptf1a* (amacrine/horizontal cells); **(F)** *otx2* (bipolar and photoreceptor cells); **(G)** *vsx1* (bipolar cell); **(H)** *prdm1a* (photoreceptor cell); **(I)** *nrl* (gray line, rod photoreceptor cell specification) and mature rod photoreceptor cell gene, *rhodopsin* (black line).

The majority of neurons produced during nervous system developmentfunction throughout the organism’s lifetime. With the exception of a few neurogenic regions that produce specific neuronal cell types, neurons that are lost due to traumatic injuries, genetic diseases or age-related disorders are not replaced in the adult human nervous system, including the retina (Kempermann et al., 2018). This inability to restore lost retinal neurons causes visual impairment and long-term blindness. In contrast to humans and other mammals, zebrafish possess an intrinsic capacity to regenerate lost retinal neurons, which is driven by resident Müller glia (Vihtelic and Hyde, 2000; Bernardos et al., 2007; Kassen et al., 2007). Although Müller glia are present in both the mammalian and zebrafish retina, damage is only sufficient to stimulate the reprogramming of zebrafish Müller glia (Bernardos et al., 2007; Kassen et al., 2007; Hoang et al., 2020), which subsequently divide asymmetrically to produce post-mitotic Müller glia and neuronal progenitor cells (NPCs; Nagashima et al., 2013). The NPCs undergo multiple rounds of cell division before they differentiate into retinal neurons (Bernardos et al., 2007). It was recently demonstrated that the arising NPCs differentiate not only into lost neurons, but also other neuronal cell types of the retina (Figure 1; Lahne et al., 2015; D’Orazi et al., 2016; Powell et al., 2016; Ng Chi Kei et al., 2017). This raises the question whether NPCs behave similar to retinal progenitors during development that produce all retinal cell types in a temporally regulated manner?

In the developing retina, multipotent progenitors pass through phases of competencies, thereby sequentially generating early and late born retinal cell types, which are governed by the temporally-regulated expression of transcription factors that either confer temporal identity or act as cell-type specific competence factors (Elliott et al., 2008; Bassett and Wallace, 2012; Mattar et al., 2015). Atoh7 is the first competence factor that is expressed in the developing retina, which is critical for producing ganglion cells (Brown et al., 2001; Kay et al., 2001; Wang et al., 2001; He et al., 2012). In the zebrafish retina, amacrine cells are subsequently specified by the expression of *ptf1a*, followed by cone photoreceptor cells (*otx2*, *crx*, *prdm1*), horizontal cells (*ptf1a*) and bipolar cells (*vsx1*, *vsx2*), while Müller glia and rod photoreceptor cells (*nrl*) belong to the last-born cell types (Nishida et al., 2003; Chow et al., 2004; Ohtoshi et al., 2004; Fujitani et al., 2006; Sato et al., 2007; Brzezinski et al., 2010; Katoh et al., 2010; Bassett and Wallace, 2012; He et al., 2012). In contrast, in the regenerating zebrafish retina, BrdU/EdU labeling approaches in combination with cell type specific differentiation markers or competence factors suggested that neurons are produced in an overlapping fashion, without following a developmental cell-type specification order (Ng Chi Kei et al., 2017; McGinn et al., 2019). However, extended exposure to thymidine analogs (three days BrdU plus four days EdU), may have prevented a temporal distinction of the onset of cell-type production (McGinn et al., 2019). Additionally, BrdU co-labeling with competence markers was investigated after proliferation had predominantly subsided following retinal damage in larval zebrafish (Ng Chi Kei et al., 2017). During retinal development, neuronal specification commences during the main proliferative phase (Li et al., 2000; He et al., 2012); hence, the period of cell fate determination during retinal regeneration may have been missed. Furthermore, developmental mechanisms might not be fully downregulated in larval retinas similar to postnatal mammalian retinas and thus, such mechanisms might influence the regenerative response (Close et al., 2006; Loffler et al., 2015). Therefore, we performed a detailed analysis of competence factor expression in NPCs, in combination with immature neuronal markers to conclude whether developmental fate specification programs are recapitulated during regeneration of the adult zebrafish retina. Understanding these processes is essential for developing strategies to induce neuronal regeneration in the diseased nervous system.

## Materials and Methods

### Fish Lines and Husbandry

Adult *albino* and transgenic *albino;Tg[atoh7:GFP]rw021* (Masai et al., 2003), *albino;Tg[ptf1a:EGFP]jh1* (Godinho et al., 2005), *albino;TgBAC[vsx1:GFP]nns5* (Kimura et al., 2008), *albino;Tg[thrb:Tomato]q22* (Suzuki et al., 2013), and *albino*;*Tg[rho:Eco.NfsB-EGFP]nt19* zebrafish (Danio *rerio*, Montgomery et al., 2010) were raised and maintained in 14 h light:10 h darkness (26.7 to 27.8°C) in the Center for Zebrafish Research, in the Freimann Life Sciences Center at the University of Notre Dame. Six to 22 months old fish of either sex (length: 2–3 cm) were used. Zebrafish were anesthetized in 1:1,000 2-Phenoxyethanol and euthanized in 1:500 2-Phenoxyethanol. The University of Notre Dame Animal Care and Use Committee approved the protocols employed in this manuscript and they are in compliance with the Association for Research in Vision and Ophthalmology statement for the use of animals in vision research.

### Damage Paradigms

#### Light Damage

Adult *albino* or transgenic *albino;Tg[atoh7:GFP]rw021*, *albino;Tg[ptf1a:EGFP]jh1*, *albino;TgBAC[vsx1:GFP]nns5* and *albino;Tg[thrb:Tomato]q22* zebrafish were dark-adapted for fourteen days and subsequently exposed to constant intense light (0, 12, 24, 36, 48, 60, 72, 84, or 96 h), as previously described (Vihtelic and Hyde, 2000; Kassen et al., 2007; Lahne et al., 2015). After 96 h of light treatment, a subset of zebrafish was allowed to recover under standard housing conditions for 2, 5, or 7 days.

To specifically damage rod photoreceptor cells, *albino*;*Tg[rho:Eco.NfsB-EGFP]nt19* zebrafish (Montgomery et al., 2010) were systemically exposed to 9 mM metronidazole, which was dissolved in system water, for 18 h at 32°C in a dark incubator. Subsequently, the metronidazole concentration was reduced to 4.5 mM and then 2.25 mM (3 h each), before zebrafish were transferred to system water devoid of metronidazole and recovered in a dark incubator at 32°C for 24, 48, 72, and 96 h. System water was exchanged daily. For determining the cell types produced following rod photoreceptor cell death, the fish were subsequently returned to standard housing conditions until 10 days of recovery (drec).

A subset of inner retinal neurons was killed by intravitreally injecting 0.5 μl of 100 mM N-Methyl-D-aspartic acid (NMDA) into adult transgenic *albino;Tg[atoh7:GFP]rw021*, *albino;Tg[ptf1a:EGFP]jh1*, *albino;TgBAC[vsx1:GFP]nns5*, and *albino;Tg[thrb:Tomato]q22* zebrafish (Powell et al., 2016; Hoang et al., 2020). Subsequently, zebrafish were maintained in system water for 0, 36, 48, 60, 72, 84, and 96 h in a dark incubator at 32°C to keep the temperature similar to that during light treatment. Zebrafish were returned to standard housing conditions at 96 h post NMDA injection and maintained up to 144 h to be consistent with the conditions applied to fish that recovered from 96 h of light treatment.

### Tissue Preservation/Preparation for Histochemical Experiments

For histochemical labeling experiments (EdU, TUNEL, immunolabeling), eyes were fixed in nine parts of 100% ethanol and one part of 37% formaldehyde at 4°C and subsequently rehydrated and cryoprotected as previously described (Lahne et al., 2015). Cryosections of 14 μm thickness were prepared and stored at −80°C until use.

To prepare flatmounts of dorsal retinas, the optic nerve was removed and the eye was cut into its dorsal and ventral hemispheres. Dorsal retinas mounted on hydrophilic PTFE cell culture inserts with the retinal pigment epithelium facing the membrane (EMD Millipore) were fixed in 4% paraformaldehyde/PBS overnight at 4°C and washed three times in PBS for 20 min at room temperature.

### EdU Labeling

Anesthetized light-damaged adult transgenic *albino;Tg[ptf1a:EGFP]jh1* and *albino; [atoh7:GFP]rw021* zebrafish were intraperitoneally injected with 50 μl of EdU (5-Ethynyl-2′-deoxyuridine, 1 mg/ml) at 24, 36, 48, 60, 72, and 84 h of light treatment (hLT) using a 32-gauge needle, as previously described (Conner et al., 2014; Lahne et al., 2015). At 96 hLT, dorsal retinal flatmounts were prepared. A subset of *albino;Tg[ptf1a:EGFP]jh1* zebrafish were additionally injected at 96 hLT and 1 drec before dorsal retinal flatmounts were fixed at 2 drec. Light-damaged *albino* zebrafish were also injected with EdU at 24, 36, 48, 60, 72, 84, and 96 hLT as well as at 1 and 2 drec (Figure 1B) and eyes were harvested at 7 drec. Anesthetized metronidazole-treated *Tg[rho:Eco.NfsB-EGFP]nt19* zebrafish were intraperitoneally injected with 50 μl of 1 mg/ml EdU at 24, 36, 48, 60, 72, 84, 96, 120, and 144 h of recovery in system water (Figure 8F) and eyes were harvested at 240 h of recovery (corresponding to the same timepoint as 7 drec following constant intense light damage). EdU integration was detected performing protocols provided by the manufacturer and was followed by immunohistochemistry.

### Immunohistochemistry

Retinal sections were immunohistochemically labeled as previously described (Vihtelic and Hyde, 2000; Kassen et al., 2007; Nelson et al., 2012; Lahne et al., 2015). Primary antibodies used in this study were rabbit anti-PCNA (1:2,000, Abcam), chicken anti-GFP (1:1,000, Abcam), mouse monoclonal anti-HuC/D (1:300, EMD Millipore), mouse monoclonal anti-Zpr-1 (1:200, ZIRC) and rabbit anti-PKCα (1:300, Santa Cruz Biotechnology) and these were detected with fluorescently conjugated secondary antibodies against mouse, rabbit or chicken (1:1,000, LifeTechnologies). The nuclear dye 4′,6-Diamidin-2-phenylindol (DAPI, 5 μg/ml, LifeTechnologies) was applied to distinguish the retinal nuclear layers.

Retinal flatmounts were incubated in chicken anti-GFP (1:1,000, Abcam), mouse anti-HuC/D (1:100, EMD Millipore) and rabbit anti-phosphorylated gap43 (1:300, Santa Cruz Biotechnology) antibodies overnight at room temperature, washed three times for 30 min in PBST, before they were exposed to fluorescently conjugated secondary antibodies against rabbit and chicken for 2 h at room temperature. Subsequently, retinal flatmounts were washed three times in PBST and once in PBS for 30 min each before they were mounted in ProlongGold (LifeTechnologies).

### Terminal Deoxynucleotidyl Transferase dUTP Nick End Labeling (TUNEL)

Prior to performing the TUNEL assay, frozen retinal sections from light-damaged *albino* zebrafish (0, 12, 24, 36, 48, 60, 72, 84, and 96 hLT) were immunohistochemically labeled for HuC/D. Following the PBS wash, slides were exposed to 4% paraformaldehyde/PBS for 20 min at room temperature to crosslink the tissue with the antibody. Subsequently, slides were washed twice in PBS for 10 min, followed by PBS containing 0.5% Triton X-100 for 15 min and were then incubated in a 1:150 dilution of 10 mg/ml proteinase K (Takara Bio) in PBS for 30 min at room temperature, followed by washes in PBST and PBS. Subsequently, the TUNEL protocol was performed as described by Thummel et al. (2010) using biotin-conjugated dNTPs (Trevigen) in combination with fluorescently labeled streptavidin (1:200, applied for 45 min at room temperature, LifeTechnologies).

### Image Acquisition and Analysis

#### Retinal Sections

Z-stack images of 6 (TUNEL) or 8 μm thickness (1024 × 1024) were acquired of the central-dorsal region of the retina on either a Nikon A1R or a C2 confocal microscope equipped with 40× plan-fluor oil immersion objectives (N.A., 1.3). Up to four channels were acquired with 405, 488, 561, and 638 nm laser lines using the “channel series” function in the Nikon Elements software to avoid spectral bleed through. Labeled cells were counted throughout the z-stack thickness and along the entire imaged retina using either Nikon Elements or Fiji software. Specifically, to determine the number of immunolabeled and/or EdU-labeled cells, the retina was subdivided into the outer nuclear layer (ONL), inner nuclear layer (INL), and ganglion cell layer (GCL) based on DAPI labeling. The few cells that were located in the inner plexiform layer were included in the INL counts. For *TgBAC[vsx1:GFP]nns5* zebrafish, cells were only considered as GFP-positive when they contained GFP within the cell, while cells that were only outlined by GFP were excluded from the counts. To assess whether inner retinal neurons were dying, retinas were subdivided into the ONL, GCL, apical INL (bipolar cells), and basal INL (amacrine cells and Müller glia), by placing a polyline along the apical boundary of HuC/D-positive amacrine cells. In a subset of experiments, the ONL was additionally subdivided into the cone and rod photoreceptor cell nuclear layers based on DAPI-labeling of nuclei (*Tg[rho:Eco.NfsB-EGFP]nt19*, *Tg[thrb:Tomato]q22*). The counts of all experiments were normalized to 300 μm length of the retina as previously described (Lahne et al., 2015) except for HuC/D counts. To determine the number of HuC/D-positive cells in the INL and GCL, a 150 μm subregion of the image was counted and the number of cells was normalized to 100 μm.

#### Retinal Flatmounts

A Nikon A1R confocal microscope equipped with a 10× plan-fluor objective (N.A., 0.3) was used to acquire z-stack images of the entire dorsal retinal flatmount using the “Large Image” tool. Higher magnification z-stacks (1024 × 1024, step-size: 0.8 μm) of the central dorsal retina were acquired using a 40× plan-fluor oil immersion objective (N.A., 1.3). Maximum intensity projections of five single z-planes at the level of the GCL or the inner plexiform layer/INL were prepared using the “ZProjection” tool in the Fiji software for *Tg[atoh7:GFP]rw021* or *Tg[ptf1a:EGFP]jh1* dorsal retinas, respectively.

### Experimental Design and Statistical Analysis

The data are presented as mean ± SE, which represent counts from at least three independent experiments (exception: Zpr-1 labeling for *thrb:Tomato* light damage timecourse, *n* = 2 rounds out of 3) and at least two biological samples per experiment [exception: round 1 of NMDA-treated *Tg[ptf1a:EGFP]jh1* (*n* = 1 for 48 and 60 h) and *TgBAC[vsx1:GFP]nns5* zebrafish (*n* = 1 for 60 and 84 h)]. Student’s *t*-test was calculated in Microsoft Excel to assess statistical significance between two treatment groups and a One-Way-ANOVA followed by Tukey’s *post hoc* test was performed for statistical comparisons between multiple treatment groups using https://astatsa.com/OneWay_Anova_with_TukeyHSD/. The n-numbers are given in the results section, the *p*-values for the ANOVA are presented in Table 1 and *p*_*Tukey*_ < 0.05 for comparisons to 0 h are indicated in the graphs. We reported all the *p*-values for *t*-tests in the results section as well as the ANOVA and Tukey’s *p*-values for comparisons of timepoints other than 0 h post damage.

**TABLE 1:** ANOVA *p*-values corresponding to the data presented in the graphs.

		ONL	INL	GCL
Light damage	*atoh7:GFP*	1.1 × 10^–16^	1.1 × 10^–16^	3.7 × 10^–11^
	*atoh7:GFP* & PCNA	2.15 × 10^–14^	1.1 × 10^–16^	5.3 × 10^–5^
	*atoh7:GFP* & HuC/D	8.0 × 10^–9^	1.1 × 10^–16^	7.3 × 10^–15^
	PCNA & HuC/D	0.2096	1.7 × 10^–11^	6.9 × 10^–8^
	*ptf1a:EGFP*	1.1 × 10^–16^	1.1 × 10^–16^	6.2 × 10^–5^
	*ptf1a:EGFP* & PCNA	3.7 × 10^–15^	1.1 × 10^–16^	0.0019
	*ptf1a:EGFP* & HuC/D	1.1 × 10^–9^	5.2 × 10^–12^	7.9 × 10^–9^
	PCNA & HuC/D	3.0 × 10^–8^	1.1 × 10^–16^	3.9 × 10^–13^
	*thrb:Tomato*	1.1 × 10^–16^	0.0008	Not determined
	*thrb:Tomato* & PCNA	1.1 × 10^–16^	0.0006	Not determined
	Zpr-1 (rod ONL)	5.1 × 10^–7^	Not determined	Not determined
	*thrb:Tomato* & Zpr-1 & PCNA	0.0007	Not determined	Not determined
	*vsx1:GFP*	4.8 × 10^–11^	Not determined	5.7 × 10^–9^
	*vsx1:GFP* & PCNA	1.6 × 10^–11^	1.1 × 10^–16^	6.5 × 10^–7^
	TUNEL	1.1 × 10^–16^	aINL: 0.003	0.11
			bINL: 0.043	
NMDA damage	*atoh7:GFP*	Not determined	6.1 × 10^–9^	Not determined
	*atoh7:GFP* & PCNA	Not determined	2 × 10^–11^	1.7 × 10^–7^
	*atoh7:GFP* & HuC/D	Not determined	2.3 × 10^–12^	6.9 × 10^–6^
	PCNA & HuC/D	Not determined	3 × 10^–12^	3.1 × 10^–5^
	*ptf1a:EGFP*	Not determined	1.7 × 10^–13^	Not determined
	*ptf1a:EGFP* & PCNA	Not determined	3.9 × 10^–9^	0.12
	*ptf1a:EGFP* & HuC/D	Not determined	2.2 × 10^–6^	0.3830
	PCNA & HuC/D	Not determined	2 × 10^–8^	0.0004
	*thrb:Tomato* (rod ONL)	3.1 × 10^–5^	Not determined	Not determined
	*thrb:Tomato* & PCNA (rod ONL)	0.0016	Not determined	Not determined
	Zpr-1 (rod ONL)	9.3 × 10^–7^	Not determined	Not determined
	*thrb:Tomato* & Zpr-1 & PCNA (rod ONL)	0.0094	Not determined	Not determined

To evaluate if expression patterns through time differed amongst the transgenes, we used a model comparison approach. All experiments displayed a bell-shaped expression pattern through time, so we chose to model expression as a Gaussian function of time. We estimated the three parameters of the Gaussian function plus the standard deviation of normally distributed errors using maximum likelihood. We also estimated parameters for a second model that included transgene identity as a factor interacting with each term in the Gaussian function. We then compared these models using a Likelihood Ratio Test. If the overall Likelihood Ratio Test indicated an effect of gene, we conducted pairwise Likelihood Ratio *post hoc* tests to determine which transgenes differed significantly from each other in terms of their expression patterns through time. Finally, time to 10% of maximum (peak) expression were calculated based on the maximum likelihood point estimates for the Gaussian function parameters to compare the onset of transgene expression across transgenes. 95% confidence intervals were calculated for time to 10% of maximum transgene expression using the Hessian matrix from the maximum likelihood parameter estimation and error propagation. Transgenes with non-overlapping 95% confidence intervals for time to 10% of maximum transgene expression were considered to differ significantly in their timing of expression. All analyses were conducted in the R Statistical Environment.

### RNA Isolation, cDNA Synthesis and Quantitative Real-Time Polymerase Chain Reaction

Dorsal or whole retinas were isolated from light-damaged *albino* zebrafish (0, 36, 48, 60, 72, 84, 96 hLT and 2, 5, and 7 drec) or metronidazole-treated *albino*;*Tg[rho:Eco.NfsB-EGFP]nt19* zebrafish (0, 24, 48, 72, 96, and 120 h after metronidazole treatment onset), respectively. Retinas were homogenized in TRIzol following the manufacturer’s instructions to the step of phase separation, when the clear phase was mixed with 75% ethanol before transferring the mixture onto a RNeasy column (Qiagen) The RNeasy kit protocol was followed starting at the step of the RNeasy column and included a 15 min DNase step (Qiagen) according to manufacturer’s instructions. The resultant RNA was stored at −80°C. cDNA was prepared using qScript cDNA super mix (QuantaBio) and subjected to quantitative real-time polymerase chain reaction (qRT-PCR) as previously described (Gorsuch et al., 2017) using primers listed in Table 2.

**TABLE 2:** Primer information.

Gene	Forward primer	Reverse primer
*18S*	5′-CGGCTACCACATCCAAGGAAGGCAGC-3′	5′-TTGCTGGAATTACCGCGGCTGCTGGCA-3′
*atoh7*	5′-ACATCATGGCCCTCAATCGG-3′	5′-AAGCGTGCAGTCACTTTCCA-3′
*nrl*	5′-CTATGCACAGCCACTCAGTCC-3′	5′-CAGCTGCTCGTCGGAGAAAC-3′
*otx2*	5′-GGCATCGGCTTGAATCCAGT-3′	5′-GCTGCTTCGGTCTCTTTTCC-3′
*pcna*	5′-TACTCAGTGTCTGCTGTGGTTTCC-3′	5′-CATTTAATAAGTGCGCCCGC-3′
*prdm1a*	5′-CTCTATGTGTGGCTGGGACC-3′	5′-ATTGTCAGCGGTGTAGGGTG-3′
*ptf1a*	5′-CCCACACAGTGACGCCTTA-3′	5′-TGAAAGAGAGTGTCCTGCGA-3′
*rho*	5′-GCTGAGCGCCACATCCA-3′	5′-AGGCACGTAGAATGCCGG-3′
*thrb*	5′-GGGTCATTTCAGGCCACGTA-3′	5′-TCGCTGACTTCATGGGCAAT-3′
*vsx1*	5′-CGTGTTTTCTCCCGAGCCA-3′	5′-ACCGGAAAGGCAGTCATCAT-3′

## Results

### RNA Expression Levels of Ganglion, Amacrine and Photoreceptor Cell Competence Factors Increased in the Light-Damaged Zebrafish Retina

In the injured zebrafish retina, Müller glia re-enter the cell cycle and divide to produce multipotent NPCs that yield most, if not all, retinal cell types independent of the cell type lost (Lahne et al., 2015; Powell et al., 2016; Ng Chi Kei et al., 2017). For example, in the light-damaged retina, ganglion, amacrine and bipolar cells are also generated besides the ablated photoreceptor cells (Figure 1A; Lahne et al., 2015; Powell et al., 2016). This suggests that NPCs in the regenerating retina behave similar to retinal progenitor cells during development, which produce different neuronal cell types in a sequential order, a process that is governed by the expression of cell specific competence factors (Bassett and Wallace, 2012; Brzezinski and Reh, 2015). To assess whether NPCs utilize developmental cell specification programs in the damaged adult zebrafish retina, we used qRT-PCR to investigate the mRNA expression levels of cell type specific competence factors.

During retinal development, ganglion cells are the first retinal neurons that are specified resulting from the expression of the transcription factor, *atoh7* (atonal basic helix loop helix transcription factor 7; Brown et al., 2001; Kay et al., 2001; Wang et al., 2001; He et al., 2012). Previously, we showed that a subset of NPCs expressed GFP under the control of the *atoh7* promoter in the light-damaged retina (Lahne et al., 2015). To confirm that *atoh7* expression is upregulated in the regenerating retina, qRT-PCR was performed using mRNA isolated from dorsal retinas at different light damage timepoints. Expression of *atoh7* transiently increased beginning at 48 h of constant light treatment (hLT) and peaking at 60 hLT (Figure 1D), before returning to baseline levels by 96 hLT (Figure 1D). In comparison, the expression of the proliferation marker, *pcna* (proliferating cell nuclear antigen) increased prior to that of *atoh7* starting at 36 hLT (Figure 1C), which represents the time when Müller glia proliferate based on previous immunohistochemical data (Kassen et al., 2007; Lahne et al., 2015). The transcription factor, *ptf1a* (pancreas associated transcription factor 1a) regulates the generation of amacrine and horizontal cells during retinal development (Fujitani et al., 2006; Nakhai et al., 2007; Jusuf and Harris, 2009; Jusuf et al., 2011). In the light-damaged retina, expression of *ptf1a* remained at baseline levels during Müller glia and early NPC proliferation at 36 and 48 hLT, respectively (Figures 1C,E). However, *ptf1a* expression sharply increased at 60 hLT and peaked at 72 hLT, before continuously decreasing to baseline levels by 5 days of recovery (drec, Figure 1E).

Photoreceptor and bipolar cells belong to a group of later born neurons, which are specified by the expression of the transcription factor, Otx2 (orthodenticle homeobox 2; Nishida et al., 2003; Sato et al., 2007). Subsequently, Vsx1 (visual system homeobox 1) and Vsx2 are transcription factors required for bipolar cell formation (Burmeister et al., 1996; Chow et al., 2004; Ohtoshi et al., 2004), while Prdm1 (PR domain containing 1a) represses the bipolar cell fate in Otx2-expressing photoreceptor precursor cells, leading to the generation of photoreceptor cells (Brzezinski et al., 2010; Katoh et al., 2010). We observed a small decline in *otx2* expression at 36 and 48 hLT relative to undamaged control retinas (0 hLT, Figure 1F), which likely corresponded to the loss of *otx2*-expressing mature photoreceptor cells in light-damaged adult retinas (Fossat et al., 2007; Housset et al., 2013). Subsequently, *otx*2 expression increased until 84 hLT, before returning to undamaged levels at 96 hLT (Figure 1F). Interestingly, at 2 drec, a time point falling within the period of rod precursor cell proliferation (Thummel et al., 2010), a second more dominant increase in *otx2* expression levels occurred (Figure 1F), which coincided with a second increase in *pcna* expression levels (Figure 1C). Although only limited *otx2* expression changes were observed, we investigated the expression of competence factors *vsx1* and *prdm1a*. The *vsx1* mRNA levels remained at baseline levels throughout the light treatment timecourse (Figure 1G), suggesting that NPCs either did not commit to the bipolar cell fate in the light-damaged retina or the numbers of bipolar cells that were produced were too low to increase *vsx1* above the expression level of mature bipolar cells in the adult retina (Chow et al., 2001). In contrast, expression levels of *prdm1a* increased significantly in a biphasic manner and remained above baseline throughout the entire regeneration timecourse investigated (Figure 1H). Unexpectedly, *prdm1a* was upregulated during Müller glia proliferation at 36 hLT, then plateaued until 60 hLT, before its expression further increased peaking at 72 and 84 hLT (Figure 1H). At subsequent timepoints, expression decreased but remained above baseline levels until 7 drec (Figure 1H). Single-cell RNA-sequencing data revealed that *prdm1a* increased in surviving cone photoreceptor cells at 10, 20, and 36 hLT, but not in Müller glia (Hoang et al., 2020), suggesting that the initial rise observed by qRT-PCR is due to increased expression in cone photoreceptor cells. Unfortunately, single-cell RNA-sequencing was not performed for timepoints during NPC proliferation and their fate specification. However, the second increase in *prdm1a* expression at a late timepoint during NPC proliferation might be related to the commitment and differentiation of NPCs into photoreceptor cells. The prolonged expression of *prdm1a* during the recovery period could relate to rod photoreceptor precursor proliferation (Figure 1H), which was previously observed at 2 drec (Thummel et al., 2010) and aligned with a secondary increase in *pcna* expression at 2 drec (Figure 1C). Therefore, we investigated the expression pattern of the rod photoreceptor cell specification factor, *nrl*, following light damage (Mears et al., 2001). The expression of *nrl* continuously decreased until 96 hLT relative to RNA-levels in undamaged controls (Figure 1I, gray line), consistent with rod photoreceptor cell loss as evidenced by simultaneously reduced *rhodopsin* expression following light damage (Figure 1I, black line). Expression of *nrl* increased at 2 drec but remained below baseline levels until 7 drec. The mRNA levels of *rhodopsin* also rose at 2 drec, but in contrast to *nrl*, returned to baseline levels by 5 drec (Figure 1I). To summarize, major competence factors (*atoh7*, *ptf1a*, *prdm1a*, and *nrl*) that drive the fate specification of all neuronal cell types, except for bipolar cells, increased expression in the light-damaged retina, suggesting that developmental fate specification programs are recapitulated.

### HuC/D Expression in *atoh7:GFP*-Positive NPCs Indicates Ganglion Cell Differentiation in the Light-Damaged Retina

The upregulation of several key competence-conferring transcription factors in light-damaged adult zebrafish retinas suggested that developmental programs are recapitulated during regeneration. However, the bipolar cell competence factor *vsx1* did not change its expression level during the regeneration timecourse, although it is known that bipolar cells are produced (Lahne et al., 2015). While qRT-PCR revealed temporal, but not spatial information, it also potentially fails to detect changes in expression of genes that are expressed in adult differentiated retinal cells, such as *vsx1* in bipolar cells. Thus, to investigate the spatial expression patterns of competence factors in the light-damaged zebrafish retina and to determine whether their expression occurs in a developmental sequence, we utilized a number of available transgenic lines (*albino;Tg[atoh7:GFP]rw021*, *albino;Tg[ptf1a:EGFP]jh1*, *albino;Tg[thrb:tomato]q22* (red cone photoreceptor cells and their precursors), *albino;TgBAC[vsx1:GFP]nns5*).

**FIGURE 2:**
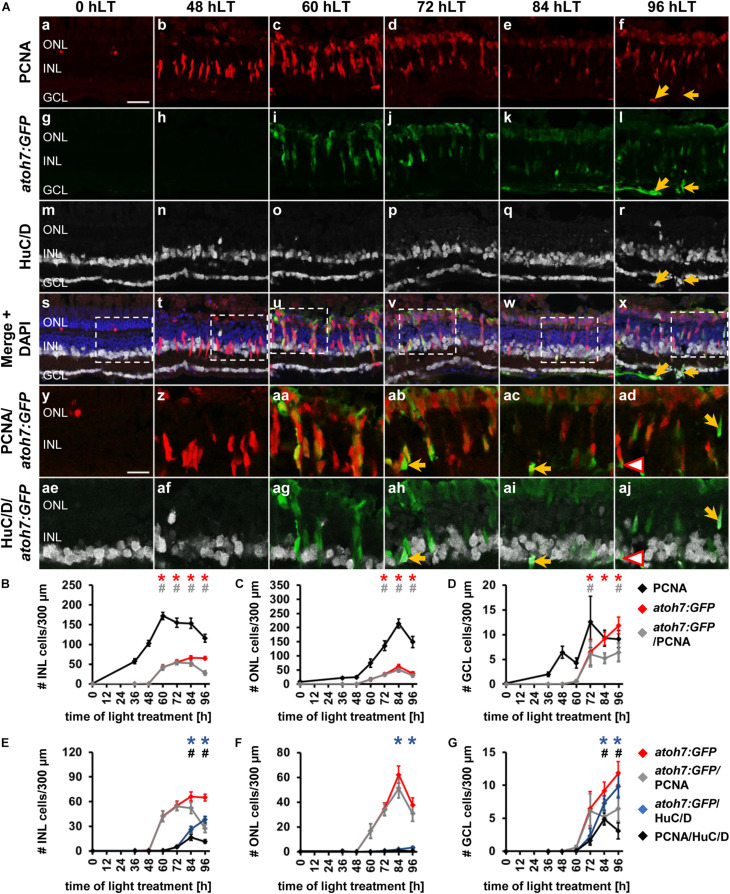
Ganglion cell competence factor *atoh7* is upregulated in the light-damaged retina. **(Aa–aj)** Single *z*-plane confocal images of retinal sections from light-damaged *Tg[atoh7:GFP]rw021* zebrafish (0, 48, 60, 72, 84, 96 hLT) immunolabeled for PCNA **(Aa–f,As–ad)**, GFP **(Ag–l,As–aj)**, HuC/D **(Am–x,Aae–aj)** and counterstained with DAPI **(As–x)**. **(Ay–aj)** Regions outlined in panels **(As–x)** at higher magnification. Yellow arrows, *atoh7:GFP*, PCNA and HuC/D-triple positive cells. Red outlined arrowheads, *atoh7:GFP* and HuC/D-double positive cell that is PCNA-negative. Scale bars, 20 μm **(Aa)** and 10 μm **(Ay)**. **(B–D)** Number of PCNA-positive, *atoh7:GFP*-positive and PCNA and *atoh7:GFP*-double positive cells in the INL **(B)**, ONL **(C)**, and GCL **(D)** over the light treatment timecourse. **(E–G)** Number of *atoh7:GFP*-positive cells and *atoh7:GFP and* PCNA*−*double positive cells in comparison to *atoh7:GFP* and HuC/D-double positive and PCNA and HuC/D-double positive cells in the INL **(E)**, ONL **(F)**, and GCL **(G)** of retinas exposed to constant intense light for 0, 36, 48, 60, 72, 84, and 96 h. Mean ± SE, *n* ≥ 12, *p_*Tukey*_ < 0.05 and ^#^*p* < 0.05 indicate comparisons to 0 hLT for the different measures that were assessed. The symbols are color-coded according to the line that they represent in the corresponding graphs (*p*_*ANOVA*_, see Table 1). Significance was not determined for PCNA in panels **(B–D)** and symbols indicating significance for *atoh7:GFP* and *atoh7:GFP* and PCNA-double-positive cells are not shown in panels **(E–G)**, as they are indicated in panels **(B–D)**. PCNA, Proliferating Cell Nuclear Antigen.

To examine ganglion cell specification we light-damaged *albino;Tg[atoh7:GFP]rw021* zebrafish and immunohistochemically labeled retinal sections for GFP, PCNA, and the ganglion/amacrine cell marker, HuC/D. In undamaged dark-adapted retinas (0 hLT), GFP driven by the *atoh7* promoter was not observed in either proliferating ONL rod precursor cells or INL cells (Figures 2Aa,g,B,C). Although increased expression of PCNA was observed in Müller glia at 36 hLT, *atoh7:GFP* was not expressed (Figures 2B–D), consistent with the qRT-PCR data. In a subset of retinal sections, a few PCNA-positive cells expressed *atoh7:GFP* at 48 hLT (0.64 ± 0.30 cells/300 μm), while the number of *atoh7*:*GFP*-positive cells increased to 42.41 ± 6.41 and 16.78 ± 5.77 in the INL and ONL, respectively, at 60 hLT (Figures 2Ac,i,u,aa,B,C,E,F; *n* = 12). Of these *atoh7:GFP*-positive cells at 60 hLT, 98.85 ± 0.46% and 96.97 ± 2.03% co-labeled with PCNA in the INL and ONL, respectively (Figures 2Ac,i,u,aa, see Table 3 for number of *atoh7:GFP*&PCNA-double positive cells; *n* = 11). At 60 hLT, only 0.55 ± 0.27 *atoh7:GFP*-positive cells/300 μm were present in the GCL (Figures 2Ai,u,D,G, *n* = 12). At subsequent timepoints (72 and 84 hLT), the number of *atoh7:GFP*-positive cells increased in the three retinal nuclear layers (Figures 2Aj,k,v,w,ab,ac,B–D), peaking in the INL and ONL at 84 hLT and in the GCL at 96 hLT. In the three nuclear layers, the majority of *atoh7:GFP*-positive cells continued to co-label with PCNA at 72 hLT (Table 3; ONL: 98.96 ± 0.54%, *n* = 15; INL: 98.44 ± 0.41%, *n* = 15; GCL: 90.88 ± 4.95%, *n* = 14), suggesting that most of the *atoh7:GFP*-positive cells remained in the cell cycle. In contrast, starting at 84 hLT, the percentage of *atoh7:GFP*-positive cells expressing PCNA was significantly reduced in the INL (Table 3; 84 hLT: 75.89 ± 7.48%, *n* = 15, *p*_*ANOVA*_ = 2.1 × 10^–8^, *p*_*Tukey*_ = 0.023; 96 hLT: 44.02 ± 7.59%, *n* = 13, *p*_*ANOVA*_ = 2.1 × 10^–8^, *p*_*Tukey*_ = 0.001) and GCL (Table 3; 84 hLT: 60.00 ± 8.23%, *n* = 15, *p*_*ANOVA*_ = 0.0015, *p*_*Tukey*_ = 0.022; 96 hLT: 47.22 ± 10.22%, *n* = 13, *p*_*ANOVA*_ = 0.0015, *p*_*Tukey*_ = 0.001) compared to 72 hLT. In contrast, the percentage of ONL *atoh7:GFP* and PCNA-double positive cells remained high and only significantly decreased at 96 hLT (Table 3; 73.98 ± 7.07%, *n* = 13, *p*_*ANOVA*_ = 0.012, *p*_*Tukey*_ = 0.014) relative to 72 hLT. These data suggested that a subset of *atoh7:GFP*-positive INL and GCL cells began exiting the cell cycle and differentiating at 84 hLT, while *atoh7:GFP*-positive ONL cells continued proliferating for a prolonged period, which could yield photoreceptor cells that are derived from an *atoh7*-lineage (Poggi et al., 2005; Feng et al., 2010).

**TABLE 3:** Quantification of transgene co-localization with PCNA, HuC/D or both in *Tg[atoh7:GFP]rw021* and *Tg[ptf1a:EGFP]jh1* in light-damaged zebrafish retinas.

Experiment	Region/Time											
	INL				ONL				GCL			
	60 hLT	72 hLT	84 hLT	96 hLT	60 hLT	72 hLT	84 hLT	96 hLT	60 hLT	72 hLT	84 hLT	96 hLT
**# *atoh7:GFP*^+^ & PCNA^+^**	41.99 ± 6.39 (12)	54.32 ± 5.34 (15)	52.17 ± 6.83 (15)	27.41 ± 4.80 (13)	16.66 ± 5.79 (12)	33.91 ± 4.77 (15)	51.18 ± 7.75 (15)	30.81 ± 6.15 (13)	0.55 ± 0.27 (12)	6.11 ± 2.57 (15)	5.22 ± 1.01 (15)	6.43 ± 1.88 (13)
**# HuC/D^+^ & PCNA^+^*(Tg[atoh7:GFP])***		4.73 ± 1.11 (15)	16.43 ± 3.76 (15)	11.42 ± 2.13 (13)						1.66 ± 0.73 (15)	4.87 ± 0.92 (15)	3.10 ± 1.21 (13)
**# *atoh7:GFP*^+^, PCNA^+^ & HuC/D^+^**		4.26 ± 1.01 (15)	16.15 ± 3.60 (15)	8.34 ± 1.86 (13)						1.59 ± 0.68 (15)	4.52 ± 0.77 (15)	2.83 ± 1.18 (13)
**# *ptf1a:EGFP*^+^ & PCNA^+^**	6.12 ± 1.35 (13)	29.17 ± 3.91 (13)	47.12 ± 5.15 (10)	26.25 ± 5.54 (11)	1.82 ± 0.58 (13)	23.12 ± 4.20 (13)	30.18 ± 5.83 (10)	7.38 ± 1.51 (11)	0 ± 0 (13)	0.27 ± 0.12 (13)	1.07 ± 0.56 (10)	0.29 ± 0.12 (11)

*Data are displayed as mean ± SE (*n*-number).*

To determine when *atoh7:GFP*-positive cells began to differentiate, we assessed the expression of HuC/D, a marker of immature and mature ganglion and amacrine cells, which is expressed within 3-6 h after the final mitosis during retinal development (Link et al., 2000; Baye and Link, 2007). Co-localization of *atoh7:GFP* with HuC/D was first observed in the INL (Figures 2Aah, arrow, E; 5.33 ± 1.21 cells/300 μm, *n* = 15) and GCL at 72 hLT (Figure 2G; 2.30 ± 1.12 cells/300 μm, *n* = 15), which coincided with the presence of a population of HuC/D- and PCNA-double positive cells in both layers (Figures 2E,G; INL: 4.73 ± 1.11 cells/300 μm; GCL: 1.66 ± 0.73 cells/300 μm; *n* = 15). In the INL and GCL, 81.45 ± 4.77% and 84.31 ± 8.36% of *atoh7:GFP* and HuC/D-double positive cells also expressed PCNA, respectively, at 72 hLT (Table 3; *n* = 15). The onset of HuC/D expression in *atoh7:GFP*-positive cells at 72 hLT was delayed by 12 h relative to the first occurrence of GFP-labeling in proliferating cells at 60 hLT (Figure 2E), which is a timeframe previously reported using RNA-sequencing analysis in the developing zebrafish retina (Xu et al., 2020). Both the number of *atoh7*:*GFP* and HuC/D-double positive and HuC/D and PCNA-co-labeled cells further increased at 84 and 96 hLT in the INL (Figure 2E and Table 3; 84 hLT: 26.34 ± 3.65 *atoh7:GFP^+^*&HuC/D^+^ cells/300 μm; *n* = 15; 96 hLT: 38.16 ± 3.65 *atoh7:GFP^+^*&HuC/D^+^ cells/300 μm, *n* = 13) and GCL (Figure 2G; 84 hLT: 7.3 ± 1.14 *atoh7:GFP^+^*&HuC/D^+^ cells/300 μm, *n* = 15; 96 hLT: 9.83 ± 1.75 *atoh7:GFP^+^*&HuC/D^+^ cells/300 μm, *n* = 13), while the percentage of triple-positive cells displayed an inverse decreasing relationship over time in the INL (Table 3; 84 hLT: 57.92 ± 7.51%, *n* = 15; 96 hLT: 25.58 ± 6.87%, *n* = 13) and GCL (Table 3; 84 hLT: 66.43 ± 8.39%, *n* = 15; 96 hLT: 28.50 ± 9.97%, *n* = 13). This suggested that *atoh7:GFP*-positive cells exited the cell cycle and differentiated into immature ganglion or amacrine cells. The majority of *atoh7:GFP* and HuC/D-double positive INL cells likely represented amacrine cells, as a subset of these are derived from an *atoh7*-lineage during retinal development (Poggi et al., 2005; Feng et al., 2010). A few *atoh7:GFP* and HuC/D-double positive cells were also present in the ONL at 84 hLT (Figure 2F; 1.98 ± 0.48 cells/300 μm, *n* = 15) and 96 hLT (Figures 2Al,r,x,F; 3.29 ± 0.82 cells/300 μm, *n* = 13). Taken together, the expression of *atoh7:GFP* in proliferating cells at 60 hLT suggested that ganglion cell specification commenced at this timepoint, while the first immature ganglion or amacrine cells were produced at 72 hLT based on *atoh7:GFP* and HuC/D co-localization.

**FIGURE 3:**
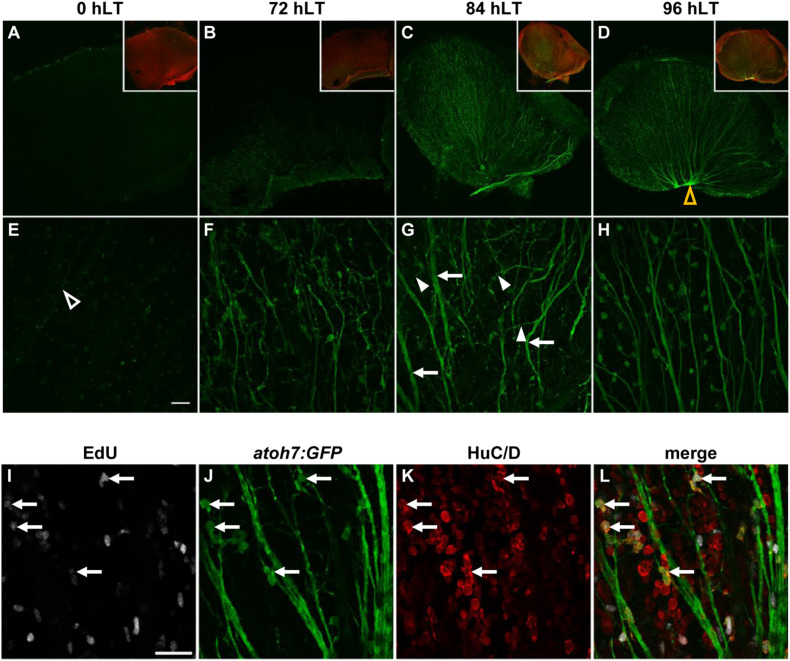
Newly generated ganglion cells extend axons. **(A–H)** Confocal images of dorsal retinal flatmounts from light-damaged *Tg[atoh7:GFP]rw021* zebrafish (0, 72, 84, 96 hLT) immunolabeled for GFP **(A–H)** and phosphorylated gap43 to identify the nerve fiber layer **(A–D**, inset) at lower **(A–D,** single *z*-plane**)** and higher magnification [**(E–H)**; maximum projections of five *z*-levels of the GCL]. Open yellow arrowhead, optic nerve head **(D)**. Open white arrowhead, thin axonal projection **(E)**. Filled arrowheads, neurites extending at an angle relative to thickened axon tracks (arrows). Scale bar, 50 μm **(E)**. **(I–L)** Single z-plane confocal images of an EdU-injected **(I,L)** *Tg[atoh7:GFP]rw021* dorsal retinal flatmount at 96 hLT immunolabeled for GFP **(J,L)** and HuC/D **(K,L)**. Arrows indicate newly generated ganglion or amacrine cells. Scale bar, 20 μm **(I)**.

To investigate whether the newly produced ganglion cells matured and developed axons that extended into the optic nerve, we examined flatmounts of dorsal retinas from light-damaged *albino*;*Tg[atoh7:GFP]rw021* zebrafish. At 0 hLT, dimly labeled *atoh7:GFP*-positive cells were observed and only a few thin axonal projections were present in the GCL/nerve fiber layer (Figures 3A,E). At 72 hLT, although only a few brightly labeled *atoh7:GFP*-positive soma were present in the GCL, many GFP-positive projections were observed that arranged in a disorganized fashion (Figures 3B,F). The low number of *atoh7:GFP*-positive soma in the GCL may suggest that mature ganglion cells began upregulating GFP; however, retinal sections revealed that INL-based *atoh7:GFP*-positive cells extended processes below the GCL and some of these bifurcated (Supplementary Figure 1), suggesting that *atoh7:GFP*-positive cells begin to generate axonal projections before their cell bodies settle into the GCL. In 84 hLT retinal wholemounts, thicker axonal projections were visible but thinner neurites, which extended in all directions continued to be present (Figures 3C,G). At 96 hLT, the majority of *atoh7:GFP*-positive neurites formed fasciculated axonal projections, which predominantly aligned in a parallel fashion and extended to the optic nerve head (Figures 3D,H, yellow open arrowhead). Importantly, a subset of these GCL-based *atoh7:GFP*-positive cells incorporated EdU when applied during the main proliferative phase, further supporting that at least a subset of newly generated *atoh7:GFP*-positive cells generated axonal projections that fasciculated by 96 hLT (Figures 3I–L). This data suggested that newly produced *atoh7:GFP*-positive cells differentiated into mature ganglion cells with axons that extended into the optic nerve head in the light-damaged retina.

### The Amacrine and Horizontal Cell Competence Factor, *ptf1a* Is Upregulated in the Light-Damaged Zebrafish Retina

**FIGURE 4:**
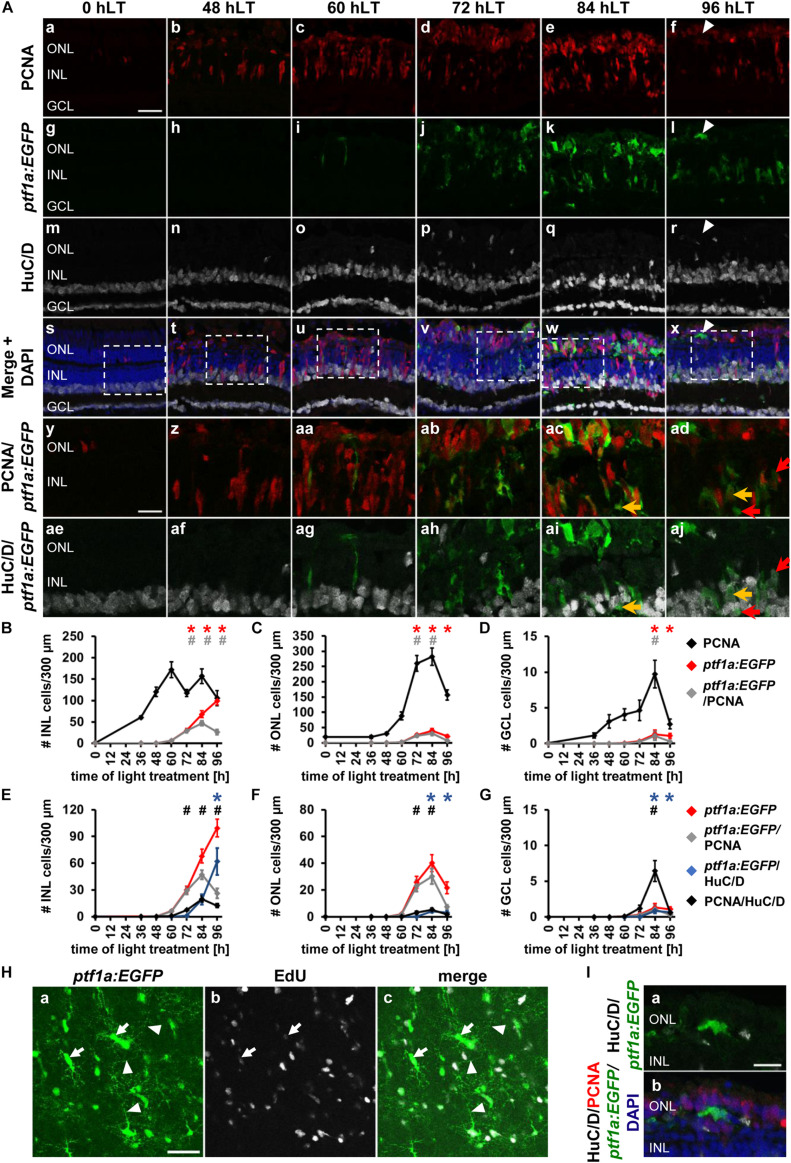
Amacrine and horizontal cell competence factor *ptf1a* is upregulated in the light-damaged retina. **(Aa–aj)** Single *z*-plane confocal images of retinal sections from light-damaged *Tg[ptf1a:EGFP]jh1* zebrafish (0, 48, 60, 72, 84, 96 hLT) immunolabeled for PCNA **(Aa–f,As–ad)**, GFP **(Ag–l,As–aj),** HuC/D **(Am–x,Aae–aj)** and counterstained with DAPI **(As–x)**. **(Ay–aj)** Regions outlined in panels **(As–x)** at higher magnification. Arrowhead, *ptf1a:EGFP*-positive ONL cell with an elongated morphology. Yellow arrows, *ptf1a:EGFP*, PCNA and HuC/D-triple positive cell. Red arrows, *ptf1a:EGFP* and HuC/D-double positive cell that is PCNA-negative. **(B–D)** Number of PCNA-positive, *ptf1a:EGFP*-positive and PCNA and *ptf1a:EGFP*-double positive cells in the INL **(B)**, ONL **(C)**, and GCL **(D)** over the light treatment timecourse. **(E–G)** Number of *ptf1a:EGFP*-positive, *ptf1a:EGFP and* PCNA*−*double positive cells in comparison to *ptf1a:EGFP* and HuC/D-double positive and PCNA and HuC/D-double positive cells in the INL **(E)**, ONL **(F)**, and GCL **(G)** of retinas exposed to constant intense light for 0, 36, 48, 60, 72, 84 and 96 h. Mean ± SE, *n* ≥ 10, *p_*Tukey*_ < 0.05 and ^#^*p* < 0.05 indicate comparisons to 0 hLT for the different measures that were assessed. The symbols are color-coded according to the line that they represent in the corresponding graphs (*p*_*ANOVA*_, see Table 1). Note, significance was not determined for PCNA in panels **(B–D)** and symbols indicating significance for *ptf1a:EGFP* and *ptf1a:EGFP* and PCNA-double-positive cells are not shown in panels **(E-G)**, as they are indicated in panels **(B–D)**. **(Ha–c)** Maximum projections of five confocal z-sections at the level of the inner plexiform/amacrine cell layer in light-damaged *Tg[ptf1a:EGFP]jh1* dorsal retinal flatmounts at 96 hLT. Newly generated *ptf1a:EGFP*-positive amacrine cells **(Ha,c)** identified by EdU [**(Hb,c)**; arrows], which was intraperitoneally injected during the proliferative phase, display neurite outgrowth (arrowheads). The images are representative of three independent experiments. **(I)** Higher magnification confocal images of the horizontally elongated *ptf1a:EGFP*-positive ONL cell at 96 hLT in panel **(Al)** (arrowhead), which potentially represents a newly generated horizontal cell. Confocal images also display HuC/D **(Ia,b)**, PCNA **(Ib)** and DAPI **(Ib)**. Scale bars, 20 μm **(Aa, Ha)** and 10 μm **(Ay, Ia)**.

The upregulation of *ptf1a* transcripts measured by qRT-PCR, suggested that amacrine and/or the developmentally later born horizontal cells were also produced in the light-damaged retina. To investigate when amacrine and/or horizontal cells were produced during zebrafish retinal regeneration, we assessed GFP expression in retinal sections from light-damaged *albino*;*Tg[ptf1a:EGFP]jh1* zebrafish. In dark-adapted undamaged zebrafish retinas (0 hLT), *ptf1a:EGFP* expression was not detected in either the INL or ONL. While PCNA-positive Müller glia and NPCs were observed at 36 and 48 hLT, respectively (Figures 4Ab,B), the first *ptf1a*:*EGFP*-expressing cells were present in the INL and ONL at 60 hLT (Figures 4Ag–i,B–D, INL: 6.24 ± 1.35 cells/300 μm; ONL: 2.10 ± 0.70 cells/300 μm; GCL: 0.06 ± 0.06 cells/300 μm, *n* = 13). The majority of these *ptf1a:EGFP*-positive cells co-labeled with PCNA (Figures 4Aaa,B–G and Table 3; INL: 97.33 ± 1.85%, *n* = 10; ONL: 91.84 ± 6.12%, *n* = 7). The number of *ptf1a:EGFP*-positive INL cells increased continuously from 72 to 96 hLT (Figures 4Aj–l,B,E; 72 hLT: 30.09 ± 3.99 cells/300 μm, *n* = 13; 84 hLT: 67.87 ± 7.78 cells/300 μm, *n* = 10; 96 hLT: 99.48 ± 9.92 cells/300 μm, *n* = 11). However, the location of *ptf1a:EGFP*-positive INL cells differed at the various timepoints. At 72 and 84 hLT, *ptf1a:EGFP*-positive cells localized throughout the thickness of the INL, while at 96 hLT, *ptf1a:EGFP*-positive cells were predominantly located in the basal INL (Figures 4Aj–l,p–r,ab–ad), consistent with their differentiation into amacrine cells.

The percentage of *ptf1a:GFP*-positive cells that co-labeled with PCNA also differed at the various timepoints. At 72 hLT, the majority of *ptf1a:EGFP*-positive INL cells co-labeled with PCNA (Figures 4Aab,B,E and Table 3 for number of *ptf1a:GFP*^+^& PCNA^+^ cells; 96.96 ± 1.19%, *n* = 13), while the percentage was significantly reduced to 75.24 ± 8.88% and 29.03 ± 5.93% of *ptf1a:EGFP*-positive cells that expressed PCNA at 84 and 96 hLT, respectively (Figures 4Aac–ad,B,E and Table 3 for numbers of *ptf1a:GFP*^+^& PCNA^+^ cells, *p*_*ANOVA*_ = 4.1 × 10^–12^; 84 hLT: *n* = 10, *p*_*Tukey*_ = 0.021; 96 hLT: *n* = 11, *p*_*Tukey*_ = 0.001). These data indicated that *ptf1a:EGFP*-positive cells began exiting the cell cycle at 84 hLT. Similar to the INL, the number of *ptf1a:EGFP*-positive ONL cells significantly increased at 72 and 84 hLT relative to 0 hLT (Figures 4Aj–k,C,F; 72 hLT: 25.80 ± 4.29 cells/300 μm, *n* = 13, *p*_*ANOVA*_ = 3.7 × 10^–15^, *p*_*Tukey*_ = 0.001; 84 hLT: 40.16 ± 6.37 cells/300 μm, *n* = 10, *p*_*ANOVA*_ = 3.7 × 10^–15^, *p*_*Tukey*_ = 0.001) but significantly decreased at 96 hLT relative to 84 hLT (Figures 4Al,C,F; 21.54 ± 4.42 cells/300 μm, *n* = 11, *p*_*ANOVA*_ = 3.7 × 10^–15^, *p*_*Tukey*_ = 0.001). Most of the *ptf1a:EGFP*-positive ONL cells co-localized with PCNA at 72 and 84 hLT (Figures 4A,C,F and Table 3; 72 hLT: 89.22 ± 3.13%, *n* = 13; 84 hLT: 72.28 ± 7.52%, *n* = 10), while they were predominantly PCNA-negative at 96 hLT (Figures 4A,C,F and Table 3; 36.52 ± 7.39%, *n* = 11). The *ptf1a:EGFP*-positive and PCNA-negative ONL cells at 96 hLT may represent horizontal cells, which are also specified by *ptf1a*. In contrast to the ONL and INL, the number of *ptf1a:EGFP*-positive GCL cells was low (Figures 4Aj–l,D,G; 72 hLT: 0.34 ± 0.16 cells/300 μm, *n* = 13; 84 hLT: 1.32 ± 0.56 cells/300 μm, *n* = 10; 96 hLT: 1.08 ± 0.35 cells/300 μm, *n* = 11), which is consistent with a limited number of displaced amacrine cells that typically localize in the GCL (Marc and Cameron, 2001).

The decreased percentage of *ptf1a:EGFP*-positive cells that co-localized with PCNA at 84 and 96 hLT, might indicate that the *ptf1a:EGFP*-positive cells exited the cell cycle and differentiated into amacrine or horizontal cells. To assess when *ptf1a:EGFP*-positive cells began differentiating, retinal sections from light-damaged *albino*;*Tg[ptf1a:EGFP]jh1* zebrafish were immunohistochemically labeled for HuC/D and PCNA. The number of *ptf1a:EGFP*-positive INL cells that expressed HuC/D was very low at 72 hLT (Figure 4E; 0.96 ± 0.24 cells/300 μm), but subsequently the number of *ptf1a:EGFP* and HuC/D-double positive cells increased (Figures 4Aai–aj,E; 84 hLT: 19.42 ± 5.81 cells/300 μm, *n* = 10; 96 hLT: 61.94 ± 15.12 cells/300 μm, *n* = 11). The increased number of *ptf1a:EGFP* and HuC/D-double positive cells at 84 and 96 hLT, together with the simultaneous decrease in PCNA expression in these cells, suggested that *ptf1a:EGFP*-positive INL cells exited the cell cycle and differentiated into amacrine cells.

To further investigate whether these newly generated *ptf1a:EGFP*-positive amacrine cells matured, we assessed whether they extended neurites and potentially integrated into the existing retinal circuit. Retinal flatmounts were prepared from light-damaged *Tg[ptf1a:EGFP]jh1* zebrafish that were intraperitoneally injected with EdU during the proliferative phase to identify newly generated amacrine cells. Maximum projections of five confocal z-sections at the level of the amacrine cell layer and the inner plexiform layer in retinal flatmounts revealed a network of thin *ptf1a:EGFP*-positive processes (Figures 4Ha,c, arrowheads). Specifically, several EdU and *ptf1a:EGFP*-double positive soma were identified that extended neurites from their cell bodies to varying degrees and that emanated into multiple directions (Figures 4Ha–c, arrows). At 2 drec, we observed EdU and *ptf1a:EGFP* double-positive cells that laminated the IPL (Supplementary Figure 2). Taken together, INL based *ptf1a:EGFP*-positive cells began to differentiate into HuC/D-positive amacrine cells at 84 hLT and displayed neurite outgrowth at 96 hLT suggesting that these cells might have integrated into the existing neuronal circuit.

The subset of *ptf1a:EGFP*-positive ONL cells that co-labeled with HuC/D increased from 0.21 ± 0.11 cells/300 μm at 72 hLT to 4.21 ± 1.03 cells/300 μm and 3.20 ± 1.07 *ptf1a:EGFP* & HuC/D-double positive cells/300 μm at 84 and 96 hLT, respectively (Figures 4F; 72 hLT: *n* = 13, 84 hLT: *n* = 10, 96 hLT: *n* = 11). Interestingly, in comparison to the INL, the percentage of *ptf1a:EGFP*-positive ONL cells that expressed HuC/D was lower at 84 hLT (INL: 25.69 ± 4.30%, ONL: 9.46 ± 1.70%, *n* = 10) and this difference became more prominent at 96 hLT (INL: 56.40 ± 10.76%, ONL: 12.36 ± 3.08%, *n* = 11). As Ptf1a is also a competence factor required for horizontal cell specification (Fujitani et al., 2006; Nakhai et al., 2007; Jusuf and Harris, 2009), it is possible that the *ptf1a:EGFP*-positive and HuC/D-negative cells represent differentiated horizontal cells. Interestingly, a subset of *ptf1a:EGFP*-positive ONL cells displayed an elongated morphology stretching along the circumferential axis of the retina similar to horizontal cells (Figures 4Al,r,x, arrowheads, I). Unfortunately, antibodies are not available that definitively identify horizontal cells in the adult zebrafish retina.

In the GCL, a small number of *ptf1a:EGFP* and HuC/D-double positive cells were present starting at 84 hLT (Figure 4G, 72 hLT: 0 ± 0 cells/300 μm, *n* = 13, 84 hLT: 0.85 ± 0.35 cells/300 μm, *n* = 10, 96 hLT: 0.65 ± 0.29 cells/300 μm, *n* = 11) and a subset of these cells expressed PCNA at 84 hLT (0.53 ± 0.31 cells/300 μm, *n* = 10). Interestingly, in *Tg[ptf1a:EGFP]jh1* zebrafish, the co-expression of HuC/D and *ptf1a:GFP* occurred subsequent to the presence of PCNA and HuC/D-double positive cells (Figure 4G; 72 hLT: 1.20 ± 0.46 cells/300 μm, *n* = 13), which suggested that ganglion cells were produced prior to amacrine cells. In support, the number of HuC/D-positive cells that expressed either *atoh7:GFP* or PCNA in *Tg[atoh7:GFP]rw021* zebrafish increased simultaneously in the GCL (Figure 2G; 72 hLT: 2.30 ± 1.12 *atoh7:GFP*^+^&HuC/D^+^ cells/300 μm, 1.66 ± 0.73 PCNA^+^&HuC/D^+^ cells/300 μm; *n* = 15). In addition, in *Tg[ptf1a:EGFP]jh1* zebrafish, the number of HuC/D- and PCNA-double positive GCL cells at 84 hLT (6.43 ± 1.47 cells/300 μm, *n* = 10) was greater than those cells expressing *ptf1a:EGFP* (0.53 ± 0.31 cells/300 μm, *n* = 10), indicating that ganglion cells were generated in greater number than *ptf1a:EGFP*-positive amacrine cells. In summary, the predominant expression of *ptf1a:EGFP* in the INL and ONL starting at 72 hLT and the subsequent co-localization with HuC/D at 84 hLT indicated that amacrine cell fate determination and differentiation occurred at these timepoints, respectively, which was subsequent to ganglion cell fate specification/differentiation, which began at 60/72 hLT. Moreover, the predominant absence of HuC/D expression in *ptf1a:EGFP*-positive ONL cells at 96 hLT, together with the elongated shape of a subset of these cells suggested that some of these *ptf1a:EGFP-*positive ONL cells became horizontal cells.

### Upregulation of the Red Cone Photoreceptor Cell Competence Factor, *thrb*, in Proliferating NPCs in the Light-Damaged Zebrafish Retina

**FIGURE 5:**
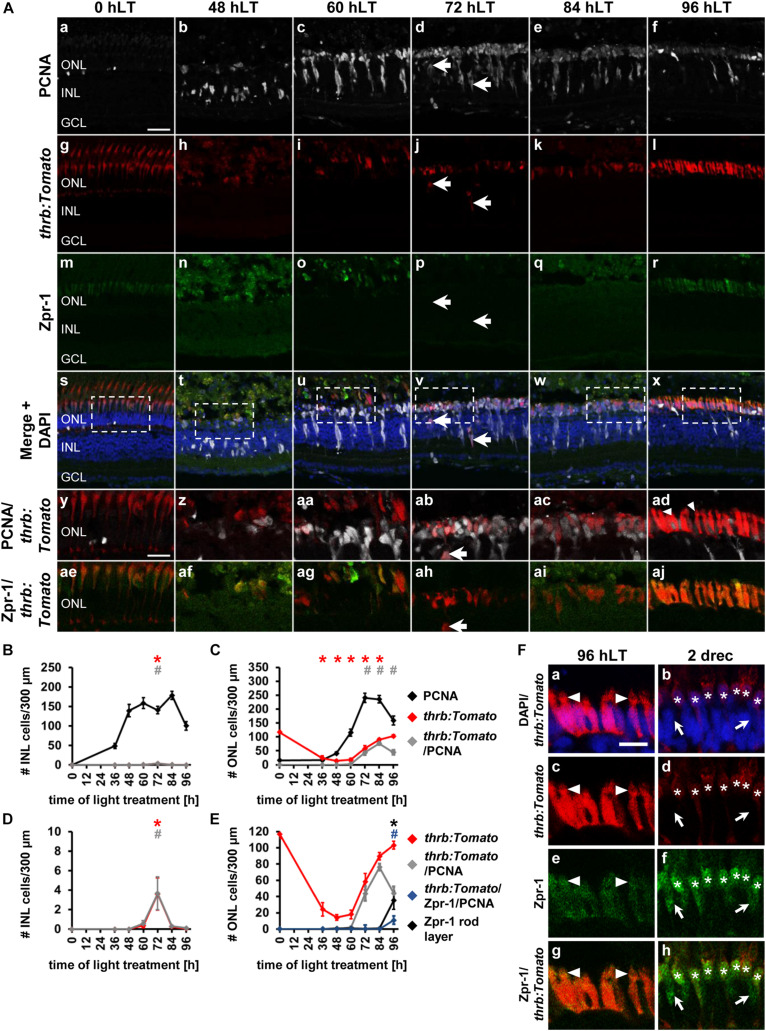
Competence factor of red cone precursor cells, *thrb*, is upregulated in the light-damaged retina. **(Aa–aj)** Single z-plane confocal images of retinal sections from light-damaged *Tg[thrb:Tomato]q22* zebrafish **(Ag–l,As–aj)**, (0, 48, 60, 72, 84, 96 hLT) immunolabeled for PCNA **(Aa–f,As–ad)** and the red/green double cone marker, Zpr-1 **(Am–x,Aae–aj)** and counterstained with DAPI **(As–x)**. **(Ay–aj)** Regions outlined in panels **(As–x)** at higher magnification. Arrows, *thrb:Tomato*-positive INL cells. Scale bars, 20 μm **(Aa)** and 10 μm **(Ay)**. **(B,C)** Number of PCNA-positive, *thrb:Tomato*-positive and PCNA and *thrb:Tomato*-double positive cells in the INL **(B)** and ONL **(C)** over the light treatment timecourse. **(D)** Number of *thrb:Tomato*-positive and *thrb:Tomato* and PCNA*-*double positive cells in the INL at a different scale to **(B)**. **(E)** Number of *thrb:Tomato*-positive, *thrb:Tomato* and PCNA-double positive cells in comparison to Zpr-1-positive cells and *thrb:Tomato*, PCNA and Zpr-1-triple positive cells in the ONL of retinas exposed to constant intense light for 0, 36, 48, 60, 72, 84 and 96 h. Mean ± SE, *n* ≥ 9, ^∗^*p*_*Tukey*_ < 0.05 and ^#^*p* < 0.05 indicate comparisons to 0 hLT for the different measures that were assessed. The symbols are color-coded according to the line that they represent in the corresponding graphs (*p*_*ANOVA*_, see Table 1). Note, significance was not determined for PCNA in panels **(B,C)** and symbols indicating significance for *thrb:Tomato*-positive and *thrb:Tomato* and PCNA-double-positive cells are not shown in panel **(E)**, as they are indicated in panel **(C)**. **(F)** Single z-plane confocal images of retinal sections from *Tg[thrb:Tomato]q22* zebrafish **(Fa–d,g,h)** co-labeled with Zpr-1 **(Fe–h)** and DAPI **(Fa,b)** at 96 hLT [cells indicated by arrowhead in panel **(Aad)** at higher zoom] and 2 drec. Arrowheads illustrate the presence of small inner/outer segments. Astericks, *thrb:Tomato* and Zpr-1 double positive cells. Arrows, Zpr-1-positive and *thrb:Tomato*-negative cells. Scale bar, 10 μm.

The qRT-PCR expression profiles of *prdm1a* suggested that cone photoreceptor cell development potentially commenced at 72 hLT. To assess the timing of cone photoreceptor cell specification in the light-damaged retina, we utilized *albino;Tg[thrb:Tomato]q22* zebrafish that express Tomato in red cone photoreceptor precursor cells during development and in mature red cone photoreceptor cells in the adult retina (Suzuki et al., 2013) and an antibody to Zpr-1, which detects arrestin 3 in red and green cone photoreceptor cells (Ile et al., 2010). In undamaged retinas (0 hLT), *thrb:Tomato* and Zpr-1 were observed in the cone photoreceptor cell layer (Figures 5Ag,m,s,y,ae,C), but not in PCNA-positive rod precursor cells in the ONL. As expected, following exposure to constant intense light, *thrb:Tomato*-positive and Zpr-1-positive cells were predominantly lost (Figures 5Ah,i,n,o,t,u,af,ag,C) and those that survived, appeared typically dysmorphic (Figures 5Ah,i,n,o,z,aa,af,ag). At 0, 36, and 48 hLT, *thrb:Tomato*-positive cells did not co-localize with PCNA (Figures 5Aa,b,g,h,s,t,y,z,B–E). In the INL, a few *thrb:Tomato*-positive cells co-localized with PCNA at 72 hLT (Figures 5Ad,j,v,ab,ah, arrows, B, D; 3.61 ± 1.66 cells/300 μm, *n* = 13), while *thrb:Tomato*-positive cells were rarely observed in the GCL (Supplementary Figure 3; 72 hLT: 2/13 retinas, 3 total cells; 84 hLT: 1/13 retinas, 1 cell). In contrast, PCNA-positive ONL cells began expressing *thrb:Tomato* at 60 hLT (Figures 5Ac,i,u,aa,ag,C,E; 2.18 ± 0.80 cells/300 μm, *n* = 13) and increased in number until 84 hLT (Figures 5Ad,e,j,k,v,w,ab,ac,C,E; 72 hLT: 43.68 ± 9.03 cells/300 μm, *n* = 11; 84 hLT: 76.28 ± 4.18 cells/300 μm, *n* = 15), before significantly decreasing at 96 hLT (Figures 5Af,l,x,ad,C,E; 44.24 ± 7.86 cells/300 μm, *n* = 14) relative to 84 hLT (Figures 5Ae,k,w,ac,C,E; 76.28 ± 4.18 cells/300 μm, *n* = 15, *p*_*ANOVA*_ = 1.1 × 10^–16^, *p*_*Tukey*_ = 0.001). The total number of *thrb:Tomato*-positive ONL cells also continuously increased from 72 to 96 hLT, when a level was reached that was not significantly different from that in the undamaged retina (Figures 5Ag,l,y,ad,C,E, 0 hLT: 116.86 ± 5.69 cells/300 μm, *n* = 13; 96 hLT: 103.04 ± 15.92 cells/300 μm, *n* = 14, *p*_*ANOVA*_ = 1.1 × 10^–16^, *p*_*Tukey*_ = 0.69). While the majority of *thrb:Tomato*-positive cells co-labeled with PCNA at 72 and 84 hLT (72 hLT: 75.96 ± 7.67%, *n* = 13; 84 hLT: 85.39 ± 2.71%, *n* = 15), only 42.35 ± 7.65% of *thrb:Tomato*-positive cells expressed PCNA at 96 hLT (*n* = 14). These data indicated that a subset of red cone precursor cells exited the cell cycle by 96 hLT.

The morphology of *thrb:Tomato*-positive cells changed from a predominantly round shape at 72 and 84 hLT (Figures 5Aab,ac) to an elongated shape at 96 hLT (Figure 5Aad). These immature *thrb:Tomato*-positive photoreceptor cells also displayed signs of compartmentalization into a cell body and inner/outer segments: *thrb:Tomato*-positive cell bodies that contained DAPI-positive nuclei (Figures 5Fa,c) and a constriction that separated DAPI-negative apical protrusions from the cell body (Figure 5F, arrowhead). These morphological changes together with reduced numbers of *thrb:Tomato*- and PCNA-double positive cells indicated that *thrb:Tomato*-positive cells had differentiated into red cone photoreceptor cells. In support of red cone photoreceptor cell differentiation at 96 hLT, Zpr-1 labeling revealed that a subset of newly generated *thrb:Tomato*-positive cells expressed Arrestin3, a marker of differentiated red and green cone photoreceptor cells at this timepoint (Figures 5Al,r,aj,E,F; 35.15 ± 11.17 *thrb:Tomato*^+^ & Zpr-1^+^ cells/300 μm, 32.46 ± 9.18% *thrb:Tomato^+^*&Zpr-1^+^/*thrb:Tomato^+^* cells, *n* = 11), but were negligible at earlier timepoints (Figures 5C,E; 72 hLT: 0.15 ± 0.10 cells/300 μm, *n* = 11; 84 hLT: 0.89 ± 0.46 cells/300 μm, *n* = 12). The presence of these *thrb:Tomato*-positive cells that expressed Arrestin3 (Zpr-1^+^) at 96 hLT demonstrates that these cells differentiated into red cones.

To determine whether red and green cone photoreceptor cell differentiation temporally differed, we examined the number of Zpr-1-positive cells that were either *thrb:Tomato*-positive or *thrb:Tomato*-negative. Interestingly, of the 43.20 ± 12.00 Zpr-1-positive cells/300 μm (*n* = 11) present at 96 hLT, nearly all of them expressed *thrb:Tomato* (42.62 ± 11.95 cells/300 μm, *n* = 11) suggesting that red, but not green, cone photoreceptor cells were produced at 96 hLT. Both the number of Zpr-1-positive cells and those co-expressing *thrb:Tomato* continued to increase at 2 drec (Zpr-1: 119.67 ± 5.25; Zpr-1 & *thrb:Tomato*: 100.01 ± 2.68, *n* = 10). However, at 2 drec we also observed Zpr-1-positive cells that lacked *thrb:tomato* expression, which significantly increased from 0.58 ± 0.34 cells/300 μm (*n* = 11) at 96 hLT to 19.66 ± 3.96 cells/300 μm (*n* = 10, *p* = 7.2 × 10^–5^) at 2 drec. These data indicated that green cone photoreceptors were produced by 2 drec, which is delayed in comparison to the generation of red cone photoreceptor cells. Taken together, these data suggested that the majority of *thrb:Tomato*-positive red cone precursor cells were positioned in the ONL, the site where photoreceptor cells are ultimately localized and that a subset exited the cell cycle and differentiated into red cone photoreceptor cells by 96 hLT, which was followed by green cone photoreceptor differentiation at 2 drec.

### The Bipolar Cell Competence Factor, *vsx1* Is Upregulated in Proliferating NPCs in the Light-Damaged Zebrafish Retina

**FIGURE 6:**
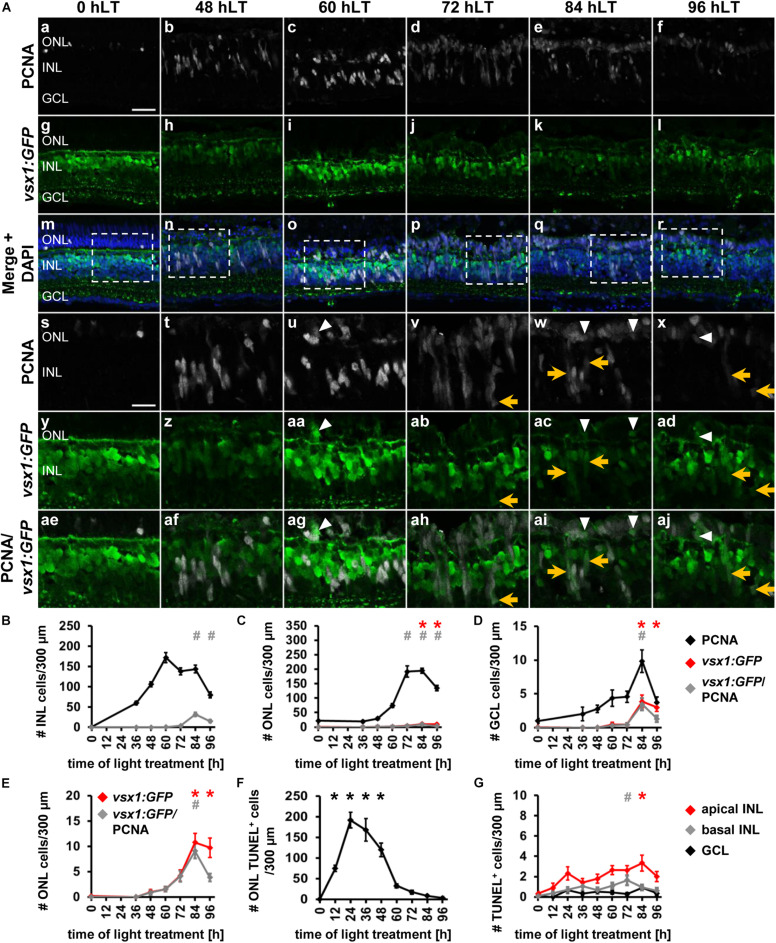
Bipolar cell competence factor *vsx1:GFP* is expressed in proliferating cells in the light-damaged retina. **(Aa–aj)** Single z-plane confocal images of retinal sections from light-damaged *TgBAC[vsx1:GFP]nns5 z*ebrafish (0, 48, 60, 72, 84, 96 hLT) immunolabeled for PCNA **(Aa–f,Am–x,Aae–aj)**, GFP **(Ag–r,Ay–aj)** and counterstained with DAPI **(Am–r)**. **(As–aj)** Regions outlined in panels **(Am–r)** at higher magnification. Scale bars, 20 μm **(Aa)** and 10 μm **(As)**. **(B–D)** Number of PCNA-positive, *vsx1:GFP*-positive and PCNA and *vsx1:GFP*-double positive cells in the INL **(B)**, ONL **(C)**, and GCL **(D)** over the light treatment timecourse. **(E)** Number of *vsx1:GFP*-positive and *vsx1:GFP* and PCNA*−*double positive cells in the ONL at a different scale. **(F,G)** Number of TUNEL-positive cells in the ONL **(F)** and in the inner retina **(G)**, (apical INL, basal INL, GCL) following constant intense light treatment for 0, 12, 24, 36, 48, 60, 72, 84 and 96 h. Mean ± SE, *n* ≥ 10. *p_*Tukey*_ < 0.05 and ^#^*p* < 0.05 indicate comparisons to 0 hLT for the different measures that were assessed. The symbols are color-coded according to the line that they represent in the corresponding graphs (*p*_*ANOVA*_, see Table 1). Note, significance was not determined for PCNA in panels **(B–D)**.

In the adult light-damaged retina, bipolar cells are also produced during the regenerative response (Lahne et al., 2015), however, the qRT-PCR data revealed unchanged *vsx1* expression during the light damage timecourse. As expression of *vsx1* in bipolar cells in the adult retina may mask the upregulation of *vsx1* in a small number of NPCs, we assessed the timing of bipolar cell specification in light-damaged *albino;TgBAC[vsx1:GFP]nns5* retinas. In undamaged zebrafish retinas, *vsx1:GFP* was expressed in mature bipolar cells located in the apical INL as previously described (Figures 6Aa,g,m,s,y; Chow et al., 2001), while only one proliferating rod precursor cell in the ONL co-localized with *vsx1:GFP* (Figures 6Aa,g,m,s,y,ae,C,E). Proliferating Müller glia at 36 hLT and NPCs at 48 hLT did not express *vsx1:GFP* (Figures 6Ab,h,n,t,z,af,B–E); however, a very small number of PCNA-positive cells co-localized with *vsx1:GFP* in the ONL at 60 hLT (Figures 6Ac,i,o,u,aa,ag, arrowhead, C, E; 1.56 ± 0.52 cells/300 μm, *n* = 14) and 72 hLT (Figures 6Ad,j,p,v,ab,ah,C,E; 4.07 ± 1.06 cells/300 μm, *n* = 14). In the INL, a few PCNA-positive cells that dimly expressed GFP were first observed at 72 hLT (Figures 6Ad,j,p,v,ab,ah, arrow, B; 3.84 ± 0.73 cells/300 μm, *n* = 14). Co-localization of PCNA and *vsx1:GFP* peaked at 84 hLT in the INL (Figures 6Ae,k,q,w,ac,ai,B; 31.66 ± 4.34 cells/300μm, *n* = 17) and ONL (Figures 6Ae,k,q,w,ac,ai,C,E; 9.07 ± 1.44 cells/300 μm, *n* = 17). Surprisingly, a small number of PCNA and *vsx1:GFP*-double positive GCL cells were also observed at 84 hLT (Figure 6D; 3.45 ± 0.89 cells/300 μm, *n* = 17). At 96 hLT, PCNA and *vsx1:GFP*-double positive cells continued to be present in both the INL (Figures 6Af,l,r,x,ad,aj,B; 15.08 ± 3.09 cells/300 μm, *n* = 17) and ONL (Figures 6Af,l,r,x,ad,aj,C,E; 3.85 ± 0.77 cells/300 μm, *n* = 17), but at significantly reduced numbers relative to 84 hLT (INL: *p*_*ANOVA*_ = 1.11 × 10^–16^, *p*_*Tukey*_ < 0.01; ONL: *p*_*ANOVA*_ = 1.55 × 10^–11^, *p*_*Tukey*_ < 0.01; *n* = 17). The expression of *vsx1:GFP* in the existing mature bipolar cells prevented us from assessing, when NPCs committed to the bipolar cell fate. However, the reduction in the number of *vsx1:GFP* and PCNA-double positive INL cells at 96 hLT relative to 84 hLT might indicate that *vsx1:GFP*-positive cells exited the cell cycle. In contrast, *vsx1:GFP*-positive cells are typically absent in the ONL and therefore, it was possible to determine whether the percentage of *vsx1:GFP*-positive cells that expressed PCNA changed during the light damage timecourse, which would indicate when cells exited the cell cycle. At 72 and 84 hLT, the majority of *vsx1:GFP*-positive cells co-localized with PCNA (72 hLT: 98.19 ± 1.41%, *n* = 14; 84 hLT: 89.25 ± 4.26%, *n* = 17). In contrast, at 96 hLT, only 50.63 ± 7.54% of the *vsx1:GFP*-positive cells expressed PCNA (*n* = 16), while the same number of *vsx1:GFP*-positive cells were present in the ONL (9.76 ± 1.93 cells/300 μm, *n* = 17) compared to 84 hLT (10.62 ± 1.76 cells/300 μm, *n* = 17), which suggested that a subset of *vsx1:GFP*-positive cells exited the cell cycle by 96 hLT. Taken together, a subset of proliferating cells expressed *vsx1:GFP* predominantly at 84 and 96 hLT in the INL and ONL, suggesting that NPCs committed to the bipolar cell fate at these timepoints. Moreover, the reduction in the percentage of *vsx1:GFP*-positive cells that co-localized with PCNA at 96 hLT indicated that NPCs fated to become bipolar cells had exited the cell cycle.

**FIGURE 7:**
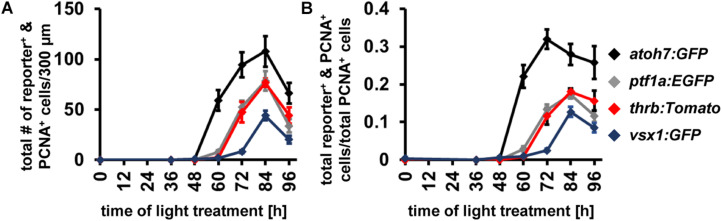
Comparison of the temporal expression patterns of neuronal competence factors in the light-damaged retina. **(A,B)** Total number (ONL, INL, and GCL combined) of *atoh7:GFP*-, *ptf1a:EGFP*-, *thrb:Tomato-* and *vsx1:GFP-*positive cells (i.e., reporter-positive cells) that express PCNA **(A)** and when normalized to the total number of PCNA-positive cells **(B)** at 0, 36, 48, 60, 72, 84, and 96 hLT. Mean ± SE, *n* ≥ 9.

Having established the expression patterns of developmental competence factors *atoh7*, *ptf1a*, *thrb* and *vsx1* in the light-damaged retina using transgenic lines, we next compared their temporal expression, adding the numbers of fluorescent reporter-positive cells that co-labeled with PCNA in the ONL, INL, and GCL for each transgene (Figure 7A). First, we performed a Likelihood Ratio test, which established an overall difference between the expression data for the different transgene-expressing PCNA-positive cells (*p* < 0.0001). A *post hoc* test between pairs of transgenes that expressed PCNA revealed that the *atoh7:GFP* expression pattern was significantly different relative to each of the other three transgenes. Both *ptf1a:EGFP* and *thrb:Tomato* expression profiles were also significantly different from *vsx1:GFP* (Figure 7A and Table 4); however, a paired comparison of *ptf1a:EGFP* and *thrb:Tomato* revealed that these were not statistically different (Figure 7A and Table 4). We obtained similar results when the number of transgene-expressing PCNA-positive cells was normalized to the number of PCNA-positive cells (Figure 7B and Table 4). While the pairwise comparisons suggested differences in transgene expression profiles, we cannot extrapolate that these are due to a difference in expression onset or expression levels. Therefore, to assess whether the onset of transgene expression in proliferating cells differed, a curve was fitted to the data and the time that corresponded to 10% of the peak expression was calculated for each transgene. Using these values, *atoh7:GFP* was initially expressed in proliferating cells at 10% of its peak at 46.78 ± 3.4 h, while *ptf1a:GFP* and *thrb:Tomato* reached 10% of its peak expression at 59.23 ± 1.89 h and 59.84 ± 2.28 h, respectively (Table 5). Comparing the upper and lower limits of the 95% confidence interval for these three transgenes suggested that *atoh7:GFP* was expressed significantly earlier in PCNA-positive cells than either *ptf1a:EGFP* and *thrb:Tomato*, which were both upregulated simultaneously (Table 5). The transgene that reached 10% of its expression peak last was *vsx1:GFP* at 69.65 ± 1.77 h, which was significantly later than the other three transgenes based on the 95% confidence interval analysis (Table 5). We performed a similar analysis for the transgene-expressing PCNA-positive cells that were normalized to the number of PCNA-positive cells and while the predicted timing at 10% peak expression changed slightly for each transgene relative to the above data (Figure 7B and Supplementary Table 1), the overall interpretation of the relative order of expression was the same. Taken together, the onset of fluorescent reporter expression differed between a subset of the transgenic lines used, but the subsequent presence of reporter-positive cells of the different transgenic lines at the same timepoints (Figures 7A,B) suggested that NPCs became competent to differentiate sequentially, but that subsequent competence factor expression overlapped in the light-damaged retina, mimicking retinal development.

**TABLE 4:** *p*-values for the overall and pairwise Likelihood Ratio Tests for comparing transgene expression in PCNA-positive cells over time.

		Light damage		NMDA	
		# transgene^+^ & PCNA^+^	# transgene^+^ & PCNA^+^/PCNA^+^	# transgene^+^ & PCNA^+^	# transgene^+^ & PCNA^+^/PCNA^+^
**p-value**	**Likelihood Ratio Test (overall)**	<0.0001	<0.0001	<0.0001	<0.0001
	***atoh7:GFP* v. *ptf1a:EGFP***	<0.0001	<0.0001	<0.0001	<0.0001
	***atoh7:GFP* v. *thrb:Tomato***	<0.0001	<0.0001	<0.0001	<0.0001
	***atoh7:GFP* v. *vsx1:GFP***	<0.0001	<0.0001	<0.0001	<0.0001
	***ptf1a:EGFP* v. *thrb:Tomato***	1	1	<0.0001	<0.0001
	***ptf1a:EGFP* v. *vsx1:GFP***	<0.0001	<0.0001	<0.0001	<0.0001
	***thrb:Tomato* v. *vsx1:GFP***	<0.0001	<0.0001	0.76	<0.0001

**TABLE 5:** Time to 10% peak expression and the corresponding lower and upper limits of the 95% confidence intervals analyzed for the number of transgene-expressing PCNA-positive cells following light or NMDA damage.

		Time to 10% peak expression [h]	S.E. × 1.96	95% Confidence interval	
				Lower	Upper
**Light damage**	***atoh7:GFP***	46.78	3.4	43.38	50.18
	***ptf1a:EGFP***	59.23	1.89	57.34	61.12
	***thrb:Tomato***	59.84	2.28	57.56	62.12
	***vsx1:GFP***	69.65	1.77	67.88	71.42
**NMDA**	***atoh7:GFP***	56.45	6.22	50.23	62.67
	***ptf1a:EGFP***	58.58	5.43	53.15	64.01
	***thrb:Tomato***	70.59	4.12	66.47	74.71
	***vsx1:GFP***	73.27	5.98	67.29	79.25

### Expression of Developmental Competence Factors in a Genetic Rod Photoreceptor Cell Ablation Model

Our data suggest that NPCs in the regenerating retina are intrinsically programmed to generate all retinal cell types in a conserved sequence, thereby mimicking NPCs during retinal development, which is consistent with the damage-induced reprogramming of Müller glia into retinal progenitor-like cells (Hoang et al., 2020). Alternatively, this phenomenon could be explained by cell death of all neuronal cell types. Previous research suggested that cell death in the light-damaged retina almost exclusively occurred in the photoreceptor cell layer (Vihtelic and Hyde, 2000; Vihtelic et al., 2006; Powell et al., 2016). However, these studies either focused on early damage timepoints or investigated cell death at one and four days post injury, while omitting intermediate timepoints. To determine whether neurons in the INL and GCL die subsequent to light damage-induced photoreceptor cell death, which could potentially stimulate the generation of inner retinal neurons, light-damaged *albino* eyes were collected every 12 h and retinal sections were labeled for HuC/D and subsequently subjected to the TUNEL assay. In agreement with previous studies, large numbers of TUNEL-positive cells were observed in the ONL (rod and cone photoreceptor cell nuclear layers combined) at 12 hLT (Figure 6F; 74.73 ± 7.45 cells/300 μm, *n* = 10) and 24 hLT (Figure 6F; peak, 191.51 ± 18.97 cells/300 μm, *n* = 12), which persisted at increased levels until 48 hLT (Figure 6F; 120.04 ± 16.52 cells/300 μm, *n* = 13) relative to control levels (Figure 6F; 0.51 ± 0.23 cells/300 μm, *n* = 11). After 48 hLT, the number of TUNEL-positive ONL cells steeply decreased to 32.31 ± 4.58 cells/300 μm at 60 hLT (Figure 6F; *n* = 12) and declined further to 3.32 ± 0.82 cells/300 μm by 96 hLT (Figure 6F; *n* = 12). To assess cell death of inner retinal neurons, the INL was subdivided into the apical and basal INL based on the position of HuC/D-labeled amacrine cells in the basal INL. Additionally, the number of TUNEL-positive cells were determined in the GCL. In all three layers, the number of TUNEL-positive cells observed were minimal throughout the light treatment timecourse (Figure 6G). As TUNEL might not detect all forms of cell death (Fricker et al., 2018), we examined the number HuC/D-positive INL and GCL cells, which were not significantly different at 36, 48, 60 and 72 hLT compared to undamaged controls (0 hLT, Supplementary Figures 4A,B; INL: *p*_*ANOVA*_ = 0.80; GCL: *p*_*ANOVA*_ = 0.74). This data together with the low number of TUNEL-positive cells suggested that only a few inner retinal neurons died in light-damaged zebrafish retinas and their cell death unlikely stimulated the generation of inner retinal neurons in the light-damaged retina.

Light-sensitive non-photoreceptor cells are present in the INL and GCL of zebrafish retinas and it is possible that light damage also induced their cell death, which might represent the few TUNEL-positive inner retinal cells (Kojima et al., 2000, 2008; Matos-Cruz et al., 2011). Tools are limited that would allow us to determine whether light-sensitive cells were dying and thereby stimulated a response in Müller glia/NPCs. However, a cell-specific genetic ablation model would allow investigating indirectly whether inner retinal cell death is necessary to stimulate the expression of developmental competence factors. To induce rod photoreceptor cell death specifically, *Tg[rho:Eco.NfsB-EGFP]nt19* zebrafish, which express *Escherichia coli* nitroreductase under the rod opsin promoter, were exposed to the prodrug metronidazole for 24 h (Montgomery et al., 2010). As previously reported, cell death occurred predominantly in the rod nuclear layer (data not shown; (Montgomery et al., 2010). In support of negligible inner retinal neuron death, we determined that the number of HuC/D-positive INL and GCL cells were not significantly different at 48, 72, and 96 h after metronidazole treatment onset (mto) relative to undamaged controls (0 h, Supplementary Figures 4C,D; INL: *p*_*ANOVA*_ = 0.26; GCL: *p*_*ANOVA*_ = 0.67).

**FIGURE 8:**
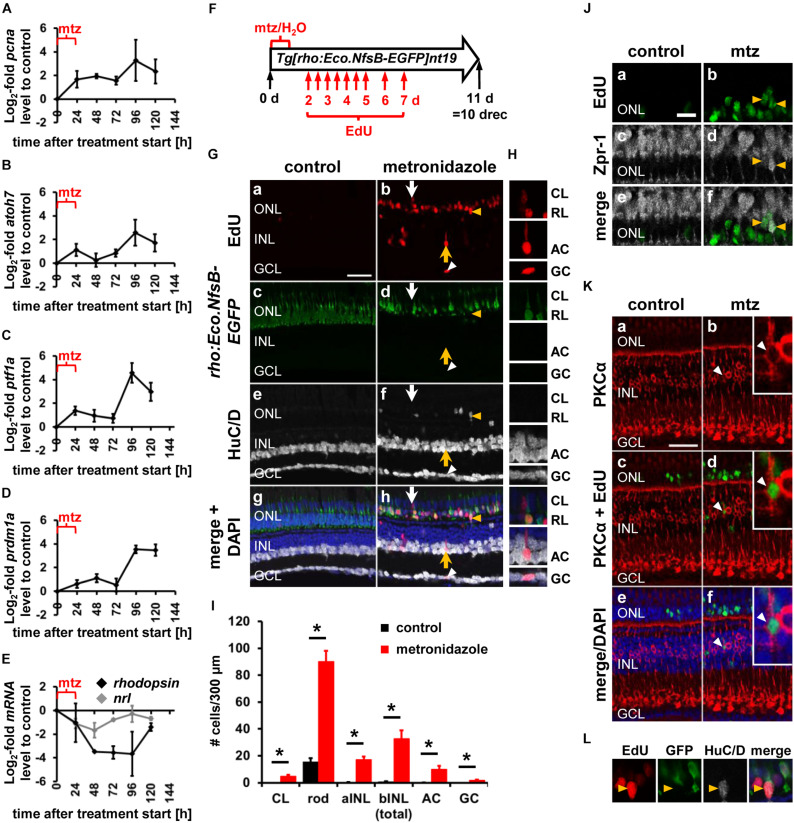
Expression of developmental competence factors and generation of all neuronal cell types following rod photoreceptor cell death in a genetic ablation model. **(A–E)** Line plots displaying mRNA expression levels expressed as log_2_-fold changes relative to 0 h controls for *pcna* **(A)**, *atoh7* **(B)**, *ptf1a* **(C)**, *prdm1a* **(D)**, *nrl* (**E**, gray line), and *rhodopsin* (**E**, black line) following metronidazole-induced rod photoreceptor cell death in *Tg[rho:Eco.NfsB-EGFP]nt19* zebrafish (24, 48, 72, 96, and 120 h after metronidazole treatment onset). Mean ± SE, *n* ≥ 3. **(F)** Schematic of the experimental paradigm: *Tg[rho:Eco.NfsB-EGFP]nt19* zebrafish were either exposed to metronidazole (mtz) or system water (H_2_O) for 24 h and subsequently recovered in system water for 10 days (10 drec). Intraperitoneal EdU injections at the indicated timepoints (red arrows). **(Ga–h,H,L)** Single *z*-plane confocal images of retinal sections from metronidazole or water-exposed EdU-injected *Tg[rho:Eco.NfsB-EGFP]nt19* zebrafish **(Ga,b,g,h)** at 10 drec that were labeled for GFP **(Gc,d,g,h)**, HuC/D **(Ge–h)**, and DAPI **(Gg,h)**. Arrowhead, GCL EdU-positive ganglion/amacrine cell; yellow arrow, INL EdU-positive amacrine cell; white arrow, EdU-positive cell in the cone nuclear layer. Scale bar, 20 μm **(Ga)**. **(H)** EdU-positive cells in panel **(Ab)** (arrows, white arrowhead) at higher magnification. **(I)** Number of EdU-positive cells in the cone nuclear layer, apical and basal INL and those identified as rod photoreceptor cells by co-labeling with *rho:Eco.NfsB-EGFP*, as amacrine and ganglion cells based on the expression of HuC/D in the basal INL or GCL, respectively, at 10 drec following exposure of *Tg[rho:Eco.NfsB-EGFP]nt19* zebrafish to either system water (control) or metronidazole for 24 h. Mean ± SE, *n* ≥ 9, Student’s *t*-test, p < 0.05. **(J,K)** Single *z*-plane confocal images from water or metronidazole-exposed EdU-injected **(Ja,b,e,f,Kc–f)** *Tg[rho:Eco.NfsB-EGFP]nt19* zebrafish at 10 drec that were labeled for Zpr-1 **(Jc–f)** or PKCα **(Ka–f)** and counterstained with DAPI **(Ke,f)**. Yellow arrowheads **(J)**, Zpr-1 & EdU-double positive cells; white arrowhead **(K)**, PKCα & EdU-positive cell. **(L)** EdU & HuC/D-double positive ONL cell (yellow arrowhead) in panel **(G)** at higher magnification. aINL, apical inner nuclear layer; bINL, basal inner nuclear layer; CL, cone nuclear layer; RL, rod nuclear layer; mtz, metronidazole.

Most of the transgenic lines we employed in previous experiments expressed GFP, which would not allow us to confidently distinguish competence factor-driven GFP from *rho:Eco.NfsB-EGFP*. Therefore, we used qRT-PCR to determine whether the expression of competence factors *atoh7*, *ptf1a*, *prdm1a* and *nrl*, which displayed prominent expression changes in the light-damaged retina when assessed by qRT-PCR (see Figure 1), were upregulated in a model in which only rod photoreceptor cells were ablated. Initially, the *pcna* expression pattern was determined. Increased expression levels of *pcna* were observed at 24 h post mto and were maintained throughout the investigated timecourse (Figure 8A). In contrast, the most prominent rise in expression levels of the ganglion cell competence factor, *atoh7*, was observed at 96 h post mto (Figure 8B). Similarly, the RNA levels of the amacrine cell and photoreceptor cell competence factors, *ptf1a* and *prdm1a*, respectively, also increased at 96 h post mto (Figures 8C,D). In contrast, RNA levels of *nrl* initially decreased (Figure 8E, gray line), which coincided with a decline in *rhodopsin* RNA levels (Figure 8E, black line) due to rod photoreceptor cell death. At 72 h post mto, *nrl* levels required for rod photoreceptor cell specification began increasing, reaching baseline levels at 96 h post mto (Figure 8E, gray line). In contrast, *rhodopsin* levels remained low at 96 h post mto and only displayed an increase toward baseline levels at 120 h post mto (Figure 8E, black line). The expression of these developmental cell fate determination factors following specific ablation of rod photoreceptor cells suggested that all neuronal cell types were generated in the genetic rod photoreceptor cell ablation model.

To determine whether all neuronal cell types were generated in the rod photoreceptor cell ablation model, *Tg[rho:Eco.NfsB-EGFP]nt19* zebrafish were exposed to system water or metronidazole for 24 h, followed by multiple intraperitoneal EdU injections (Figure 8F). In undamaged retinas, EdU-positive cells were predominantly present in the rod photoreceptor cell nuclear layer of the ONL (Figures 8Ga,c,g; 85.63 ± 4.96% of total EdU-positive cells = 23.56 ± 3.51 cells/300 μm, *n* = 21). Co-labeling of EdU and GFP revealed that 15.55 ± 2.61 cells/300 μm of the 23.56 ± 3.51 ONL-based EdU-positive cells/300 μm represented *rho:Eco.NfsB-EGFP*-positive rod photoreceptor cells (Figure 8I). As previously described, EdU-positive cells were absent in the cone photoreceptor cell layer and negligible in the apical INL (Figures 8Ga,c,g,I; 0.24 ± 0.13 cells/300 μm, *n* = 21) and basal INL (Figures 8Ga,c,g,I; 0.81 ± 0.16 cells/300 μm, *n* = 21). In the GCL, EdU-positive cells were present but none of these co-labeled with HuC/D suggesting that ganglion/amacrine cells were not produced in undamaged central zebrafish retinas during persistent neurogenesis (Figure 8I). Following rod photoreceptor cell death, the number of EdU-positive cells was significantly increased in all the regions/cell types that were assessed compared to the corresponding regions/cell types in the undamaged controls (Figures 8G,H,I). In the rod photoreceptor cell nuclear layer, 114.57 ± 9.40 EdU-positive cells/300 μm were present and 89.97 ± 8.08 of these EdU-positive cells/300 μm co-expressed *rho:Eco.NfsB-EGFP* (Figure 8I; *n* = 9). While the EdU-positive cells in the rod photoreceptor cell nuclear layer continued to represent the majority of EdU-positive cells (66.28 ± 3.71%) in the metronidazole-exposed retina, EdU-positive cells were also observed in the cone photoreceptor cell layer (Figures 8G, white arrow), H, I; metronidazole: 4.93 ± 0.83 cells/300 μm, *n* = 9; control: 0 ± 0 cells/300 μm, *n* = 21, *p* = 5 × 10^–10^) and a subset of these co-labeled with the red/green cone marker Zpr-1 (Figure 8J). EdU-positive cells were also present in the apical INL (Figures 8G,I; metronidazole: 17.17 ± 2.24 cells/300 μm; *n* = 9; control: 0.24 ± 0.13 cells/300 μm, *n* = 21, *p* = 2.7 × 10^–12^) with some corresponding to PKCα-positive bipolar cells (Figure 8K), the basal INL (Figures 8G,H,I; metronidazole: 32.63 ± 6.13 cells/300 μm; *n* = 9 control: 0.81 ± 0.16 cells/300 μm, *n* = 21, *p* = 7.9 × 10^–9^) and GCL (metronidazole: 3.23 ± 0.81 cells/300 μm; *n* = 9 control: 0.51 ± 0.15 cells/300 μm, *n* = 21, *p* = 3.2 × 10^–6^). In the basal INL and GCL, 10.00 ± 2.34 and 1.77 ± 0.56 EdU-positive cells/300 μm co-labeled with the amacrine and ganglion cell marker, HuC/D, respectively (Figures 8G, yellow arrow and white arrowhead, respectively, H, I). Similar to the light-damaged retina, we also observed newly generated HuC/D-positive cells in the ONL (Figures 8G,L). Importantly, however, these EdU and HuC/D-double positive cells did not co-express *rho:Eco.NfsB-EGFP* (Figure 8L). Taken together, this data showed that developmental cell fate determination factors were also expressed in an ablation model that only affected rod photoreceptor cells and that these factors drove the generation of cells in all retinal nuclear layers. This data indicated that NPCs were intrinsically programmed to produce all neuronal cell types, but mechanisms might be in place that favor the generation of rod photoreceptor cells following their specific death in *Tg[rho:Eco.NfsB-EGFP]nt19* zebrafish.

### Expression of Developmental Competence Factors in the NMDA-Damaged Retina

Having established that developmental neuronal competence factors are also expressed following light damage-induced photoreceptor cell death in a sequence that is temporally similar to retinal development, we next investigated whether the sequence is also recapitulated following inner retinal cell death. Inner retinal neurons were ablated by injecting zebrafish intravitreally with the glutamate receptor agonist, NMDA and cell death was assessed using TUNEL and quantifying the number of HuC/D-positive ganglion and amacrine cells and the number of Zpr-1-positive red/green cones. At 36 h post NMDA exposure, 9.67 ± 3.90 TUNEL-positive cells/300 μm (*n* = 9) were present in the GCL, which was significantly increased relative to undamaged controls (Supplementary Figure 4E, 1.13 ± 0.32 cells/300 μm, *p* = 0.044; *n* = 9). The increased number of TUNEL-positive cells correlated with a significant reduction in the number of HuC/D-positive GCL cells at 36 h post NMDA (Supplementary Figure 4F; 36 h: 34.88 ± 6.04 cells/300 μm, *n* = 9; 0 h: 57.19 ± 5.05 cells/300 μm, *n* = 8, *p*_*ANOVA*_ = 0.040, *p*_*Tukey*_ = 0.024). While there were also fewer HuC/D-labeled GCL cells at 48, 60 and 72 h post NMDA, this effect was not statistically significant (Supplementary Figure 4F; *p*_*ANOVA*_ = 0.04, 48 h: *p*_*Tukey*_ = 0.30, 60 h: *p*_*Tukey*_ = 0.35, 72 h: *p*_*Tukey*_ = 0.077). In the basal INL, the number of TUNEL-positive cells also significantly increased from 1.29 ± 0.41 cells/300 μm in undamaged retinas to 9.18 ± 2.58 cells/300 μm at 36 h post NMDA (Supplementary Figure 4E; *n* = 9, *p* = 0.008). While fewer HuC/D-positive cells were present at 36 h post NMDA than at 0 h, this reduction was not statistically significant (Supplementary Figure 4G; 36 h: 83.69 ± 1.88, *n* = 9, 0 h: 95.22 ± 5.70, *n* = 8, *p*_*ANOVA*_ = 0.11). Similarly, the number of HuC/D-positive cells were not statistically different at subsequent timepoints (Supplementary Figure 4G; *p*_*ANOVA*_ = 0.11). We also assessed TUNEL in the ONL and observed a small, but significant, increase in the number of TUNEL-positive rod ONL cells at 36 h compared to undamaged retinas (36 h: 4.34 ± 1.19 cells/300 μm, *n* = 9; 0 h: 0.93 ± 0.30 cells/300 μm, *n* = 9, *p* = 0.014). In contrast, the numbers of TUNEL-positive cells in the cone nuclear layer or apical INL were not statistically different relative to the controls (cone layer: 36 h: 0.24 ± 0.24 cells/300 μm, *n* = 9; 0 h: 0.10 ± 0.1 cells/300 μm, *n* = 9, *p* = 0.58; aINL, 36 h: 1.24 ± 0.42, *n* = 9, 0 h: 0.55 ± 0.31 cells/300 μm, *n* = 9, *p* = 0.20). In support of NMDA not affecting cone survival, the number Zpr-1-positive red and green cone photoreceptor cells was similar at 0, 36, 48, 60, 72 and 84 h post NMDA treatment (Supplementary Figure 4H; *p*_*ANOVA*_ = 0.26, *n* = 9 (0, 36, 60 h), *n* = 8 (48, 72 h), *n* = 10 (84 h). This data suggests that NMDA treatment primarily affects GCL and bINL cells.

**FIGURE 9:**
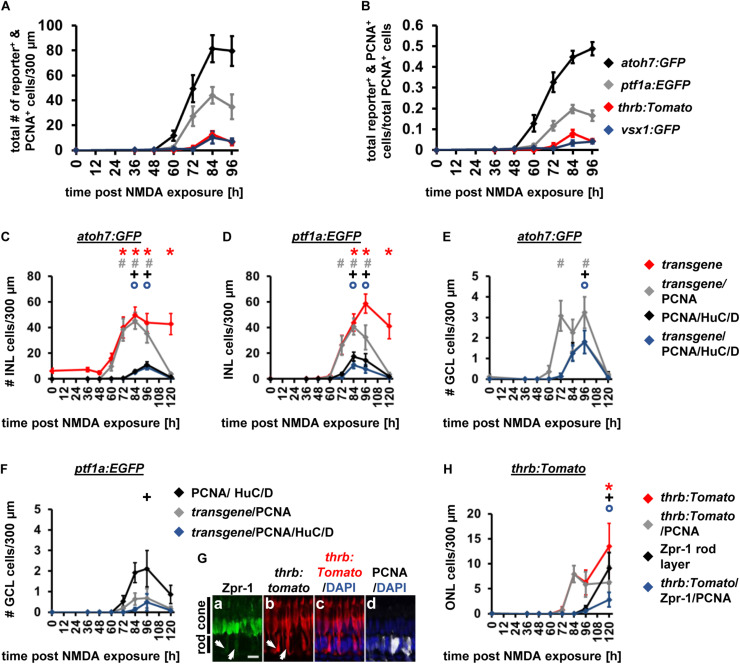
Comparison of the temporal expression patterns of neuronal competence factors in the NMDA-damaged retina. **(A,B)** Total number (ONL, INL, and GCL combined) of *atoh7:GFP-*, *ptf1a: EGFP-, thrb:Tomato-* and *vsx1:GFP-*positive cells (i.e., reporter-positive cells) that express PCNA **(A)** and when normalized to the total number of PCNA-positive cells in NMDA-damaged retinas **(B)**. Mean ± SE, *n* ≥ 7. **(C,D)** Number of transgene-positive cells, transgene-positive cells that express PCNA and those that are triple-positive for the transgene, PCNA and HuC/D as well as the number of HuC/D and PCNA-positive cells in the INL of NMDA-exposed *Tg[atoh7:GFP]rw021* **(C)** and *Tg[ptf1a:EGFP]jh1* zebrafish retinas [**(D)**; 0, 36, 48, 60, 72, 84, 96, and 120 h post NMDA exposure]. **(E,F)** Number of transgene-positive cells that express PCNA and those that also co-localize with HuC/D as well as the number of HuC/D and PCNA-positive cells in the GCL of NMDA-exposed *Tg[atoh7:GFP]rw021* **(E)** and *Tg[ptf1a:EGFP]jh1* zebrafish retinas **(F)**. Mean ± SE, *n* ≥ 9. **(G)** Single confocal z-stack images of *Tg[thrb:tomato]q22* retinas **(Gb,c)** at 120 h post NMDA exposure labeled for Zpr-1 **(Ga)**, PCNA **(Gd)**, and DAPI **(Gc,d)**. Arrows, Zpr-1 and *thrb:tomato*-double positive cells in the rod photoreceptor cell nuclear layer. Scale bar, 10 μm **(Ga)**. **(H)** Number of Zpr-1-positive cells, *thrb:Tomato*-positive cells, those that co-labeled with PCNA or those triple-positive for *thrb:Tomato*, PCNA and Zpr-1 in the rod ONL of NMDA-exposed retinas. Mean ± SE, *n* ≥ 8, *p_*Tukey*_ < 0.05, ^#^*p* < 0.05, ^+^*p* < 0.05, and °*p* < 0.05 indicate comparisons to 0 h post NMDA for the different measures that were assessed. The symbols are color-coded accordingto the line that they represent in the corresponding graphs (*p*_*ANOVA*_, see Table 1).

Having established a cell death profile, we next injected NMDA intravitreally into either *Tg[atoh7:GFP]rw021*, *Tg[ptf1a:EGFP]jh1*, *Tg[thrb:Tomato]q22* or *TgBAC[vsx1:GFP]nns5* zebrafish to assess the relative sequence of transgene expression in proliferating cells. We applied the same Likelihood Ratio Test that we used to statistically analyze the light damage data sets. The Likelihood Ratio Test revealed that the data is explained by a complex model suggesting overall differences in the expression profiles of the different transgenes expressed in proliferating cells over time (*p* < 0.0001). Subsequent *post hoc* analysis for pair-wise comparisons indicated that the expression profiles significantly differed over time for all paired combinations except for *thrb:tomato* and *vsx1:GFP* (Figure 9A and Table 4). As these comparisons cannot give insight into whether the onset of transgene expression differed, we fitted curves to each of the transgene data sets and determined the predicted time at 10% of peak expression and compared the confidence intervals (Table 5). The 10% of peak transgene expression for *atoh7:GFP* was reached at 56.45 ± 6.22 h and for *ptf1a:EGFP* approximately two h later at 58.58 ± 5.43 h (Table 5). While there was a small shift in expression onset, the confidence intervals overlapped indicating that the observed time difference was not statistically significant (Table 5). In contrast, the time to 10% of peak expression of either *thrb:Tomato* (70.59 ± 4.12 h) or *vsx1:GFP* (73.27 ± 5.98 h) in proliferating cells was significantly later relative to either *atoh7:GFP* or *ptf1a:EGFP* (Table 5). However, the time to 10% of peak of *thrb:Tomato* expression in proliferating cells did not significantly differ to that of *vsx1:GFP* (Table 5). Normalizing the number of transgene-expressing PCNA-positive cells to the number of PCNA-positive cells, we observed distinctly different curves for *atoh7:GFP* and *ptf1a:GFP* (Figure 9B) and therefore we also applied the Likelihood Ratio (*p* < 0.0001, Supplementary Table 1) and determined the time at 10% of peak expression for this data set. The *atoh7:GFP*-expressing PCNA-positive cells that were normalized to the number of PCNA positive cells reached 10% of peak expression at 50.34 ± 6.28 h (Supplementary Table 1), approximately 7 h before the normalized number *ptf1a:EGFP-*expressing PCNA-positive cells expressed at 10% to peak (57.89 ± 4.09 h, Supplementary Table 1). While this temporal expression difference of approximately 7 h was greater than that observed for the number of transgene-expressing PCNA-positive cells (∼2 h), the overlap of the 95% confidence intervals between *atoh7:GFP* and *ptf1a:EGFP* suggested that this difference was not statistically significant (Supplementary Table 1). The time to 10% of peak expression was significantly later for the normalized *thrb:tomato*-expressing PCNA-positive cells (68.5 ± 3.53 h, Supplementary Table 1) relative to *atoh7:GFP* and *ptf1a:EGFP.* In contrast, the time at 10% to peak expression could not be confidently determined for the normalized *vsx1:GFP*-expressing PCNA-positive cells (63.51 ± 72.96 h, Supplementary Table 1) as the peak of expression cannot be confidently predicted. To summarize, while statistically we cannot distinguish the onset of *atoh7:GFP* and *ptf1a:EGFP*, represented by the time to 10% of peak expression, it is clear that the *atoh7* transgene was expressed prior to *ptf1:EGFP* based on identifying more *atoh7:GFP-*positive cells than *ptf1a:EGFP-*positive cells at 60 hLT (Figures 9A,B). Additionally, these two transgenes were upregulated significantly earlier than both *thrb:Tomato* and *vsx1:GFP*.

Interestingly, comparing the fraction of transgene- and PCNA-double positive cells of the total number of PCNA-positive cells between NMDA- and light-damaged retinas revealed differences and similarities for the different transgenic lines. The fraction of *atoh7:GFP* and PCNA-double positive cells of the total number of PCNA-positive cells increased in NMDA-damaged retinas relative to light-damaged retinas, when the peak of expression was compared (Figures 7B, 9B; NMDA 96 h: 0.49 ± 0.03, *n* = 13, light damage 72 hLT: 0.31 ± 0.02, *n* = 15, *p* = 8.5 × 10^–5^). Surprisingly, the fraction of PCNA-positive cells that expressed GFP driven by the amacrine cell competence factor *ptf1a*, that specifies the cell type that was predominantly lost following NMDA exposure, was similar in both damage paradigms (Figures 7B, 9B; NMDA 84 h: 0.20 ± 0.02, *n* = 12; light-damage 84 hLT: 0.17 ± 0.01, *n* = 10, *p* = 0.33). In contrast, the fraction of *thrb:Tomato* and PCNA-double positive cells of the total number of PCNA-positive cells was significantly reduced in NMDA- relative to light-damaged retinas (Figures 7B, 9B; NMDA 84 h: 0.08 ± 0.02, *n* = 10; light damage: 84 hLT: 0.18 ± 0.01, *n* = 15, *p* = 5.9 × 10^–6^), which was consistent with the absence of photoreceptor cell loss following NMDA exposure (Powell et al., 2016). Reduced levels were also observed for *vsx1:GFP* in NMDA-damaged retinas compared to light-damage (Figures 7B, 9B; NMDA 96 h: 0.04 ± 0.01, *n* = 11; light damage 84 hLT: 0.13 ± 0.01, *n* = 17, *p* = 6.8 × 10^–5^). Thus, differences in the fraction of PCNA-positive cells that express the different fluorescent reporters when compared between the NMDA and light damage model suggested that competence factor expression and consequently cell fate decisions were regulated to a certain degree according to the cell type lost.

Downstream of fate specification, we determined the timing of differentiation, co-labeling retinal sections from *Tg[atoh7:GFP]rw021* and *Tg[ptf1a:EGFP]jh1* zebrafish with the differentiation marker, HuC/D. At 72 h post NMDA exposure, a few HuC/D and PCNA-double positive cells were observed in a subset of *Tg[atoh7:GFP]rw021* retinal sections (Figure 9C, 0.37 ± 0.18 cells/300 μm, *n* = 11). At subsequent timepoints (84 h, 96 h), the number of HuC/D-positive INL cells expressing PCNA increased (Figure 9C; 84 h: 5.87 ± 1.17 cells/300 μm, *n* = 13; 96 h: 11.11 ± 2.22, *n* = 13). 5.87 ± 1.17 cells/300 μm (*n* = 13) and 9.12 ± 1.91 cells/300 μm (*n* = 13) of HuC/D and PCNA-double positive cells, co-localized with *atoh7:GFP* at 84 and 96 h post NMDA exposure, respectively (Figure 9C), which corresponded to 91.89 ± 3.26% at 84 h and 79.83 ± 5.11% at 96 h. In NMDA-damaged *Tg[ptf1a:EGFP]jh1* zebrafish, a few HuC/D and PCNA-double-positive cells were present at 72 h (Figure 9D; 3.93 ± 1.08 cells/300 μm, *n* = 12) and these increased at 84 h (Figure 9D; 17.45 ± 3.36 cells/300 μm, *n* = 12). Only 32.37 ± 10.10% (*n* = 10) and 51.59 ± 9.82% (*n* = 11) of HuC/D and PCNA-double positive INL cells co-localized with *ptf1a:EGFP* at 72 and 84 h, respectively (Figure 9D), which suggested that the *ptf1a:EGFP*-negative proportion of cells might begin to differentiate into ganglion cells in the INL at the same time as *ptf1a:EGFP*-positive amacrine cells. In the GCL of *Tg[atoh7:GFP]rw021* zebrafish, a few HuC/D and PCNA expressing cells were present at 84 h post NMDA exposure (Figure 9E; 1.33 ± 0.44 cells/300 μm, *n* = 13) and similar to the INL, the majority of these co-localized with *atoh7:GFP* (Figure 9E; 97.91 ± 2.08%, *n* = 8, 1.25 ± 0.39 cells/300 μm, *n* = 13), suggesting that ganglion cells were produced at this timepoint. In support, only 0.48 ± 0.41 *ptf1a:EGFP* and PCNA-double positive cells/300 μm expressed HuC/D (*n* = 12), while 2.11 ± 0.89 HuC/D and PCNA-double positive cells were present in the GCL at the same timepoint (Figure 9F), indicative of the predominant production of ganglion cells in the GCL prior to the differentiation of displaced amacrine cells.

We also examined when *thrb:Tomato*-positive red cone precursor cells differentiated using Zpr-1. As photoreceptor cells were not lost following NMDA exposure, the presence of *thrb:Tomato* and Zpr-1-positive cells was assessed in the rod ONL, which was the site of ONL proliferation (Figure 9G). While *thrb:Tomato*-positive cells that expressed PCNA were first observed in the rod ONL at 84 h post NMDA exposure (Figure 9H; 7.93 ± 1.7 cells/300 μm, *n* = 10), co-localization with Zpr-1 predominantly occurred at 120 h post NMDA exposure (Figure 9H; 96 h: 0.56 ± 0.4 cells/300 μm, *n* = 12; 120 h: 2.8 ± 1.48 cells/300 μm, *n* = 10). At this timepoint, a total of 9.12 ± 3.09 Zpr-1-positive cells/300 μm (Figure 9H; *n* = 10) were present in the rod ONL, showing that only 27.25 ± 8.90% (*n* = 9) of Zpr-1-positive cells co-expressed PCNA and *thrb:Tomato*. However, all the Zpr-1-positive cells (9.12 ± 3.09 cells/300 μm) in the rod ONL expressed *thrb:Tomato* (9.12 ± 3.09 cells/300 μm) at 120 h post NMDA exposure, suggesting that the Zpr-1-positive cells differentiated into red cone photoreceptor cells, while green cone photoreceptor cells were not produced at this timepoint. In contrast, at 144 h post NMDA, 51.93 ± 11.44% of Zpr-1-positive cells in the rod ONL were *thrb:tomato*-negative, which corresponded to 5.65 ± 3.09 cells/300 μm. This suggested that green cone photoreceptors were produced subsequent to red cone photoreceptors following NMDA-exposure, which is similar to the light-damaged retina. To summarize, in the NMDA-damaged retina, ganglion cells were produced prior to amacrine cells in the GCL and red cone photoreceptor cell differentiation was delayed relative to ganglion and amacrine cell differentiation, which mimicked the sequence of differentiation marker expression observed in the light-damaged retina.

## Discussion

We demonstrate that expression of the developmental competence factors, *atoh7, ptf1a*, and *prdm1a*, required for generating ganglion, amacrine, and photoreceptor cells, increased following photoreceptor loss. Moreover, fluorescent reporters under promotor control of the competence factors *atoh7*, *ptf1a*, *thrb*, and *vsx1* were expressed in a subset of NPCs with a temporal onset of expression predominantly mimicking the developmental fate specification sequence: (1) *atoh7:GFP*, (2) *ptf1:EGFP* and lastly, *vsx1:GFP*. The expression of *thrb:Tomato* shifted from a timing similar to *ptf1:EGFP* following light damage to that of *vsx1:GFP* after NMDA exposure. Expression of neuronal maturation markers in transgene expressing cells further supported that ganglion cell generation commenced prior to amacrine cells, which was followed by red cone photoreceptor cells. Additionally, amacrine and ganglion cells displayed morphological features of maturation, such as neurite/axon outgrowth while red cone photoreceptors compartmentalized to form inner/outer segments.

This is the first study showing that the amacrine, red cone precursor, and bipolar cell competence factors *ptf1a*, *thrb*, and *vsx1*, respectively, were initially expressed in NPCs in response to retinal damage, while *atoh7:GFP* expression in NPCs was previously reported by several groups (Fimbel et al., 2007; Lahne et al., 2015; Ng Chi Kei et al., 2017). The unchanged *atoh7*, *ptf1a*, and *vsx1* expression levels between 0 and 36 hLT measured by qRT-PCR and the very low read counts observed in a recent bulk RNA-sequencing study that investigated gene expression changes during Müller glia reprogramming (Hoang et al., 2020) are in agreement with the absence of transgene expression from these gene promoters in Müller glia. Importantly, the expression of the different developmental competence factors in NPCs was consistent with generating all neuronal cell types independent of those that were ablated (Lahne et al., 2015; Powell et al., 2016; Ng Chi Kei et al., 2017). In light-damaged retinas, intense light exposure might destroy photosensitive inner retinal neurons and consequently induced competence factor expression that regulated regeneration of inner retinal neurons (Kojima et al., 2008; Matos-Cruz et al., 2011). However, only a low number of TUNEL-positive INL and GCL cells were present following light damage, in agreement with previous studies (Vihtelic and Hyde, 2000; Vihtelic et al., 2006; Powell et al., 2016). Moreover, selective rod photoreceptor cell damage in a genetic ablation model (Montgomery et al., 2010) induced developmental competence factor expression and the generation of the different neuronal cell types. This approach, together with the very low number of TUNEL-positive cells following light damage, indicated that loss of inner retinal neurons unlikely stimulated the expression of amacrine or ganglion cell competence factors. Rather, expression of the same competence factors in NPCs during development and regeneration suggests that cell fate specification mechanisms are conserved. In support, RNA-sequencing analysis during early stages of Müller glia reprogramming in either the light or NMDA-damaged retina (0, 4, 10, 20, 36 hLT) revealed that the gene expression signature of reprogrammed zebrafish Müller glia following damage is similar to retinal progenitor cells during development (Hoang et al., 2020). This suggests that Müller glia reactivate developmental programs that likely initiate a sequential cascade of competence factor expression in NPCs. Similar RNA-sequencing approaches at later timepoints of the regeneration response will be necessary to identify in more detail the gene regulatory networks that drive the different stages of cell fate specification and differentiation.

The specification of retinal cell types in a sequential, but overlapping, order during development, results in ganglion cell generation first, followed by amacrine cells and then simultaneously bipolar and photoreceptor cells, before horizontal cells are produced last in zebrafish (Young, 1985; He et al., 2012). To assess the temporal sequence of transgene expression onset in the regenerating retina, we fitted a Gaussian function of time to the different transgene data sets and determined the time at 10% to peak expression. The onset of *atoh7:GFP* expression occurred, based on the time at 10% to peak expression, prior to *ptf1a:EGFP* and *thrb:Tomato* expression, which were upregulated in a similar timeframe but significantly earlier than *vsx1:GFP* in NPCs, which suggests that cell types are produced in a sequential manner. During development, the timing of *thrb:Tomato* expression in relation to *ptf1a:EGFP* was examined in the context of horizontal cell precursors, but not amacrine cells (Suzuki et al., 2013). However, *crx:CFP* (photoreceptor and bipolar cells) was expressed shortly before *ptf1a:EGFP* expression increased in postmitotic amacrine cells during retinal development (Shen and Raymond, 2004; Almeida et al., 2014). Thus, expression of *thrb* in red cone precursor cells prior to their final mitosis (Suzuki et al., 2013) suggests that *thrb:Tomato* expression alongside *ptf1a:GFP* in the light-damaged retina most likely represents the developmental expression sequence.

In NMDA-damaged retinas, *atoh7:GFP* and *ptf1a:EGFP* were expressed in NPCs at 10% to peak expression at 56.45 ± 6.22 and 58.58 ± 5.43 h, respectively and, while the difference (∼7 h) was more pronounced when the transgenes-expressing NPCs were normalized to the number of proliferating cells, the overlap in the 95% confidence intervals of both transgenes suggested that the predicted expression onset is not distinct. However, we observed more *atoh7:GFP*-positive than *ptf1a:EGFP*-positive NPCs at 60 h following NMDA. This may suggest that more complex models are necessary to fit the data to demonstrate whether their expression onset is statistically distinct. Alternatively, if we analyzed timepoints more frequently than 12 h, it is possible that we would identify a statistical difference. Interestingly, in NMDA-damaged retinas, *thrb:Tomato* expression onset was predicted at 70.59 ± 4.12 h, which was close to the expression onset of *vsx1:GFP* (73.27 ± 5.98), rather than the observed onset alongside *ptf1a:GFP* in light-damaged retinas. This later *thrb:Tomato* expression in the NMDA- relative to the light-damaged retina might be regulated by similar mechanisms that caused a small delay in *vsx1:GFP* expression when early born ganglion or amacrine cells were absent in developing *atoh7* mutants or *ptf1a* morphants, respectively (Kei et al., 2016). In contrast to the sequential expression of competence factors in NPCs in the light- and NMDA-damaged retinas, *atoh7*, *ptf1a* and *prdm1a* were upregulated at the same time, when RNA was assessed from metronidazole-treated *Tg[rho:nsfB.Eco-EGFP]* zebrafish. However, this experiment was designed to determine whether or not these factors are expressed in a retina in which only rod photoreceptors were ablated, rather than their expression onset. A more detailed temporal analysis and approaches that allow investigation at the cellular level will be necessary in the future to address this question in a genetic ablation model.

The reduced fraction of proliferating *thrb:Tomato-*positive cells in NMDA relative to light-exposed retinas further suggests regulation of cell type production in a damage-dependent manner, similar to previous reports (Fraser et al., 2013; Powell et al., 2016; D’Orazi et al., 2020). Surprisingly, the fraction of *ptf1a:EGFP*-positive NPCs that yields amacrine cells did not change between light- and NMDA-damaged retinas, although NMDA induced death of a subset of amacrine cells (Powell et al., 2016). As amacrine cells belong to a group of earlier born neurons, it is possible that NPCs are intrinsically programmed to produce a fixed proportion of amacrine cells independent of the cell type lost. In support, photoreceptor and bipolar cell, but not amacrine cell, production was significantly increased within *atoh7* morphant clones that developed in wild-type retinas (Boije et al., 2015). Interestingly, similar feedback mechanisms regulating cell fate specification in the developing retina might interact with damage stimuli in the regenerating retina, where a subset of cells is lost. The signaling mechanisms that fine tune cell type specification in the adult regenerating retina according to the cell type lost, require further investigation in the future. This knowledge is ultimately crucial to efficiently induce proper neuron production following stimulation of Müller glia proliferation in the damaged mammalian retina (Karl et al., 2008; Yao et al., 2016).

The transcription factor Atoh7 is required for ganglion cell differentiation during retinal development (Brown et al., 2001; Kay et al., 2001; Wang et al., 2001). However, Atoh7-positive cells give rise to a lineage that produces ganglion, amacrine and photoreceptor cells, and to a lesser extent bipolar and horizontal cells (Poggi et al., 2005). Thus, we cannot infer that *atoh7:GFP* expression directly correlates with ganglion cell production. However, co-labeling of the differentiation marker, HuC/D with *atoh7:GFP* prior to that with *ptf1a:EGFP*, which is expressed after the final mitosis in cells that produce amacrine cells, indicates that ganglion cells differentiated before amacrine cells in the light-damaged retina. Furthermore, red cone photoreceptor cells differentiated subsequent to ganglion and amacrine cells, based on Zpr-1 expression in *thrb:Tomato*-positive cells at 96 hLT, which approximated the timing previously reported in light-damaged retinas (Bernardos et al., 2007). Interestingly, the absence of Zpr-1-positive cells lacking *thrb:Tomato*-expression at 96 hLT and their subsequent presence at 2 drec indicated that red cone photoreceptor cells differentiated before green cone photoreceptor cells. This observation is in agreement with fish retinal development, where cone photoreceptor subtypes are generated in a specific order, with red cones differentiating prior to green cones (Stenkamp et al., 1996, 1997; Schmitt et al., 1999; Raymond and Barthel, 2004). GFP driven by *vsx1*, which is a transcription factor necessary for bipolar cell differentiation, but also expressed at low level in progenitor cells during development (Chow et al., 2004; Ohtoshi et al., 2004; Vitorino et al., 2009), was the last transgene that was upregulated in the light-damaged retina. The onset of *vsx1:GFP* expression at 72 hLT, a time when a subset of *atoh7:GFP*-positive cells express HuC/D, supports that bipolar cells are produced subsequent to ganglion cell generation. However, without other definitive bipolar cell differentiation markers we cannot predict the exact timing of bipolar cell differentiation.

Previously, EdU/BrdU labeling revealed that bipolar and HuC/D-positive ganglion and amacrine cells were produced simultaneously suggesting an absence of a developmental birth order in ouabain-damaged retinas (McGinn et al., 2019). While the temporal overlap of transgene expression in NPCs agrees with a simultaneous production of neuronal types, our detailed analysis of the onset of competence factor expression in combination with cell type specific differentiation markers supports that cell type generation commenced sequentially. A second study utilizing different damage models in larval *Tg[atoh7:GFP]* and *Tg[vsx1:GFP]* zebrafish combined with BrdU-tracing concluded that fate specification was regulated dynamically without following the developmental birth order (Ng Chi Kei et al., 2017). Specifically, following poke injury of larval retinas, *atoh7:GFP* was expressed in BrdU-positive cells subsequent to *vsx1:GFP* expression, while in a genetic model that ablates amacrine/horizontal cells only *vsx1:GFP* co-labeled with BrdU during the period investigated. We also observed differences in a damage-dependent manner, however, *atoh7:GFP* was expressed prior to *vsx1:GFP* in both the NMDA- and light-damaged retinas, suggesting that the developmental differentiation sequence was predominantly mimicked in both adult light and NMDA-damaged retinas. This raises the question how these differences in the cell fate determination sequence between damaged larval and adult retinas can be explained. Because Ng Chi Kei et al. (2017) assessed transgene expression in BrdU-positive cells several days after proliferation had subsided, fate specification might have been missed. Our data show that the onset of ganglion cell fate specification occurred before the peak of proliferation and that specification of the different cell types was only shifted by h similar to zebrafish development (He et al., 2012). Alternatively, programmed cell death occurs in developing embryonic and larval retinas, which is thought to finetune retinal tissue architecture and circuitry (Biehlmaier et al., 2001). Thus, it is possible that cells that are typically programmed to die are instead maintained to partially compensate for the cell loss experienced in the damaged larval retina. Such adjustments in cell maintenance could also provide feedback mechanisms that might result in the dynamic regulation of cell fate determination but also reduce the need to regenerate neurons in damaged larval retinas. It will be necessary to decipher the mechanisms that underlie the differences in cell fate determination in larval versus adult retinas in the future.

The newly generated supernumerary amacrine and ganglion cellsdisplayed neurite outgrowth into the IPL and extension of fasciculating axons to the optic nerve head, respectively, by 96 hLT suggesting that these cells morphologically mature. Additionally, similar to previous reports, HuC/D-positive cells ectopically localized in the ONL and IPL in the regenerated retina (Lahne et al., 2015; Ranski et al., 2018). It remains to be determined whether these additionally-produced ganglion and amacrine cells and those that ectopically localize following light-damage are maintained long-term and whether these cells form functional circuits. Elimination of overproduced cells is supported by a drastic decrease in the number of BrdU-labeled cells from 4 to 7 days post-injury (Fausett and Goldman, 2006). However, at least a subset of newly generated amacrine/ganglion cells were maintained at 30 days post light damage (Powell et al., 2016). Similarly, a subset of new retinal neurons persisted at 11 days post TNFα- and γ-secretase inhibitor-induced reprogramming of Müller glia in undamaged retinas (Conner et al., 2014). Regenerated retinal cones and bipolar cells established synapses with surviving horizontal and cone photoreceptor cells, respectively (D’Orazi et al., 2016; Yoshimatsu et al., 2016; McGinn et al., 2018) and vision functionally recovered by 98 days post-ouabain damage (Sherpa et al., 2008). However, it is unknown whether newly generated supernumerary amacrine and ganglion cells form functional synaptic connections with existing and/or regenerated neurons in the light-damaged retina and whether these positively or negatively influence the visual response. To develop strategies that regenerate neurons from an endogenous source with the aim to functionally recover human vision in the future, it will also be critical to understand whether ectopically located neurons influence visual output in the regenerated retina.

## Data Availability Statement

The original contributions presented in the study are included in the article/Supplementary Material, further inquiries can be directed to the corresponding author.

## Ethics Statement

The animal study was reviewed and approved by Office of Research IACUC Committee, University of Notre Dame.

## Author Contributions

ML conceived the study and prepared the manuscript. ML and MB performed the experiments and analyzed the data. SJ performed statistical analysis on some of the data and assisted in the presentation of these statistical analyses. ML and DH edited the manuscript. All authors contributed to the article and approved the submitted version.

## Conflict of Interest

The authors declare that the research was conducted in the absence of any commercial or financial relationships that could be construed as a potential conflict of interest.

## Supplementary Material

Supplementary Figure 1 A subset of INL *atoh7:GFP*-positive cells extend processes below the ganglion cell layer. **(A–C)** Maximum projections of nine confocal images of *Tg[atoh7:GFP]rw021* retinal sections immunolabeled for GFP **(A,C,D)**, HuC/D **(B–D)**, and PCNA **(D)** at 72 hLT. Yellow arrows indicate *atoh7:GFP-positive* soma located in the INL that extend processes below HuC/D-positive ganglion cells (arrowhead).

Supplementary Figure 2 Neurite extension into the IPL at 2 drec. **(A–L)** Confocal images from an EdU-injected **(B–D,F–H,J–L)** *Tg[ptf1a:GFP]jh1* retinal wholemount immunolabeled for GFP **(A,C–E,G–I,K,L)** and DAPI **(D,H,L)**. **(A–D)** Single z-plane confocal images. **(E–L)** 3D XY **(E–H)** and XZ Maximum projection **(I–L)** of *ptf1a:GFP* and EdU double-positive cell displayed in **(A–D)**. To visualize the neurites in the XY view the GCL and lower part of the IPL were removed. Arrowheads indicate neurite in the IPL. Scalebar, 5 μm.

Supplementary Figure 3 Expression of *thrb:Tomato* is negligible in the GCL. Line graphs displaying the number of PCNA, *thrb:Tomato* and PCNA and *thrb:tomato*-double positive cells in the GCL of light-damaged *Tg[thrb:tomato]q22* zebrafish retinas. Mean ± SE, n ≥ 9.

Supplementary Figure 4 Damage paradigms differentially affect inner retinal neuron survival. **(A–D)** Histograms displaying the number of HuC/D-positive cells in the INL **(A,C)** and GCL **(B,D)** following light-damage **(A,B**; 0, 36, 48, 60, 72 hLT**)** and metronidazole exposure of *Tg[rho:Eco.nsfB-EGFP]nt19* zebrafish **(C,D**; 0, 48, 72, 96 h post mto**)**. **E)** TUNEL-positive cells in the undamaged retina and at 36 h after NMDA exposure in the rod and cone nuclear layers, the apical and basal INL and the GCL. **(F–H)** Histograms displaying the number of HuC/D-positive cells in the GCL **(F)** and INL **(G)** and the number of Zpr-1-positive cells **(H)** in NMDA-damaged retinas. Mean ± SE, n > 8. p*_*t*_*_–test_ < 0.05 in **E**, *p*_*ANOVA*_ = 0.04, *p*_*Tukey*_ < 0.05 in **F**. aINL, apical inner nuclear layer, bINL, basal inner nuclear layer; GCL, ganglion cell layer.
