# Supplementary figures and images for: The Regenerating Adult Zebrafish Retina Recapitulates Developmental Fate Specification Programs
Source: Front Cell Dev Biol. 2021 Feb 1;8:617923. doi: 10.3389/fcell.2020.617923 (PMC7882614; doi:10.3389/fcell.2020.617923)

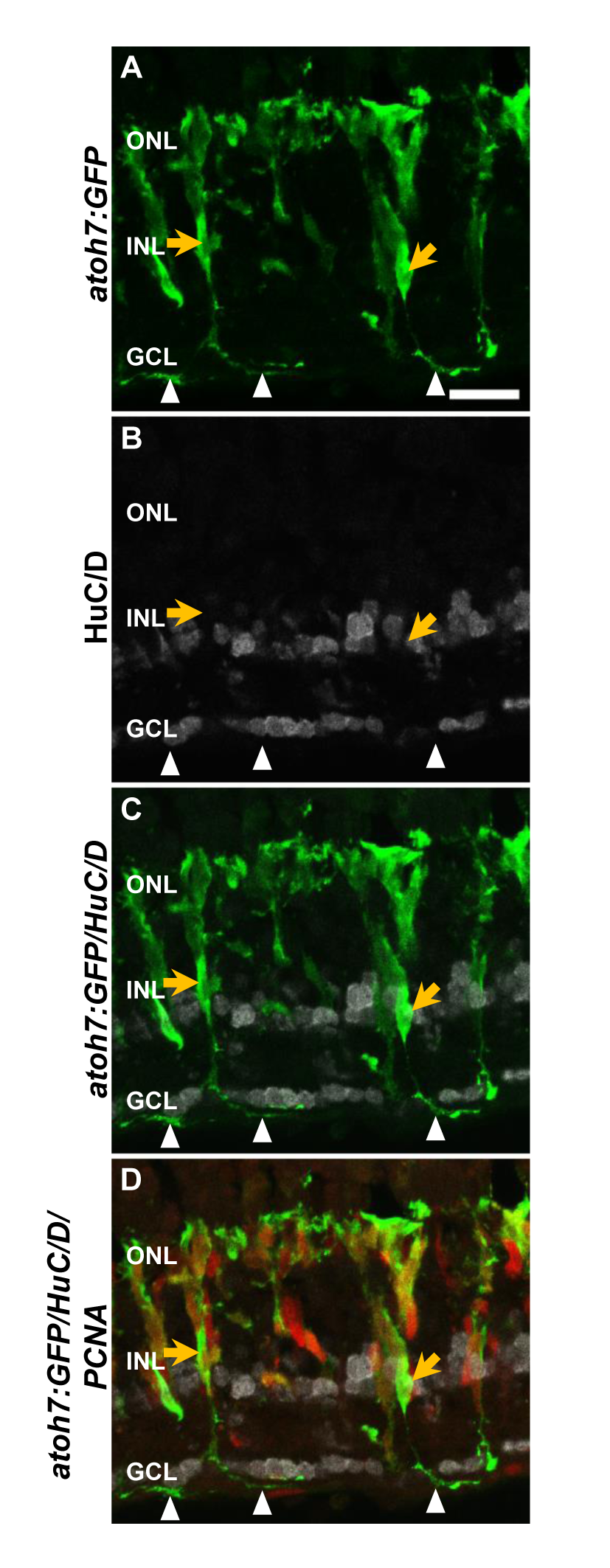

Supplement: Supplementary Figure 1 — A subset of INL atoh7:GFP-positive cells extend processes below the ganglion cell layer. (A–C) Maximum projections of nine confocal images of Tg[atoh7:GFP]rw021 retinal sections immunolabeled for GFP (A,C,D), HuC/D (B–D), and PCNA (D) at 72 hLT. Yellow arrows indicate atoh7:GFP-positive soma located in the INL that extend processes below HuC/D-positive ganglion cells (arrowhead). [file Image_1.tiff]

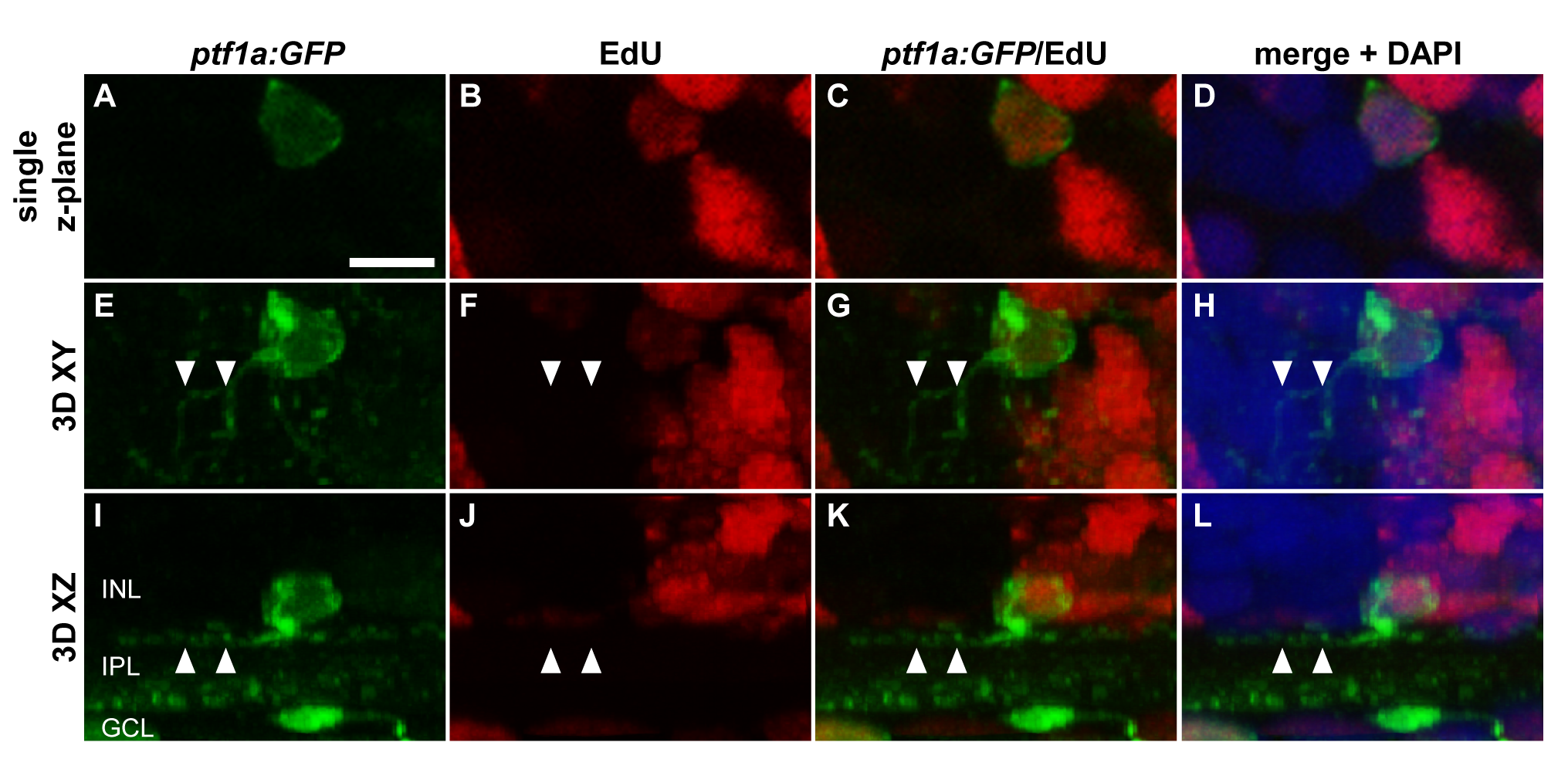

Supplement: Supplementary Figure 2 — Neurite extension into the IPL at 2 drec. (A–L) Confocal images from an EdU-injected (B–D,F–H,J–L) Tg[ptf1a:GFP]jh1 retinal wholemount immunolabeled for GFP (A,C–E,G–I,K,L) and DAPI (D,H,L). (A–D) Single z-plane confocal images. (E–L) 3D XY (E–H) and XZ Maximum projection (I–L) of ptf1a:GFP and EdU double-positive cell displayed in (A–D). To visualize the neurites in the XY view the GCL and lower part of the IPL were removed. Arrowheads indicate neurite in the IPL. Scalebar, 5 μm. [file Image_2.TIF]

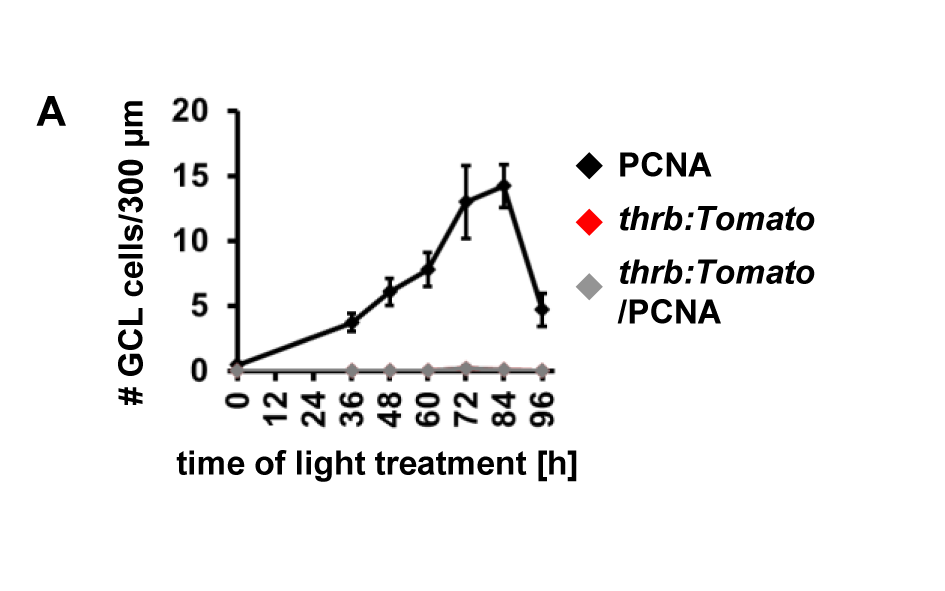

Supplement: Supplementary Figure 3 — Expression of thrb:Tomato is negligible in the GCL. Line graphs displaying the number of PCNA, thrb:Tomato and PCNA and thrb:tomato-double positive cells in the GCL of light-damaged Tg[thrb:tomato]q22 zebrafish retinas. Mean ± SE, n ≥ 9. [file Image_3.TIF]

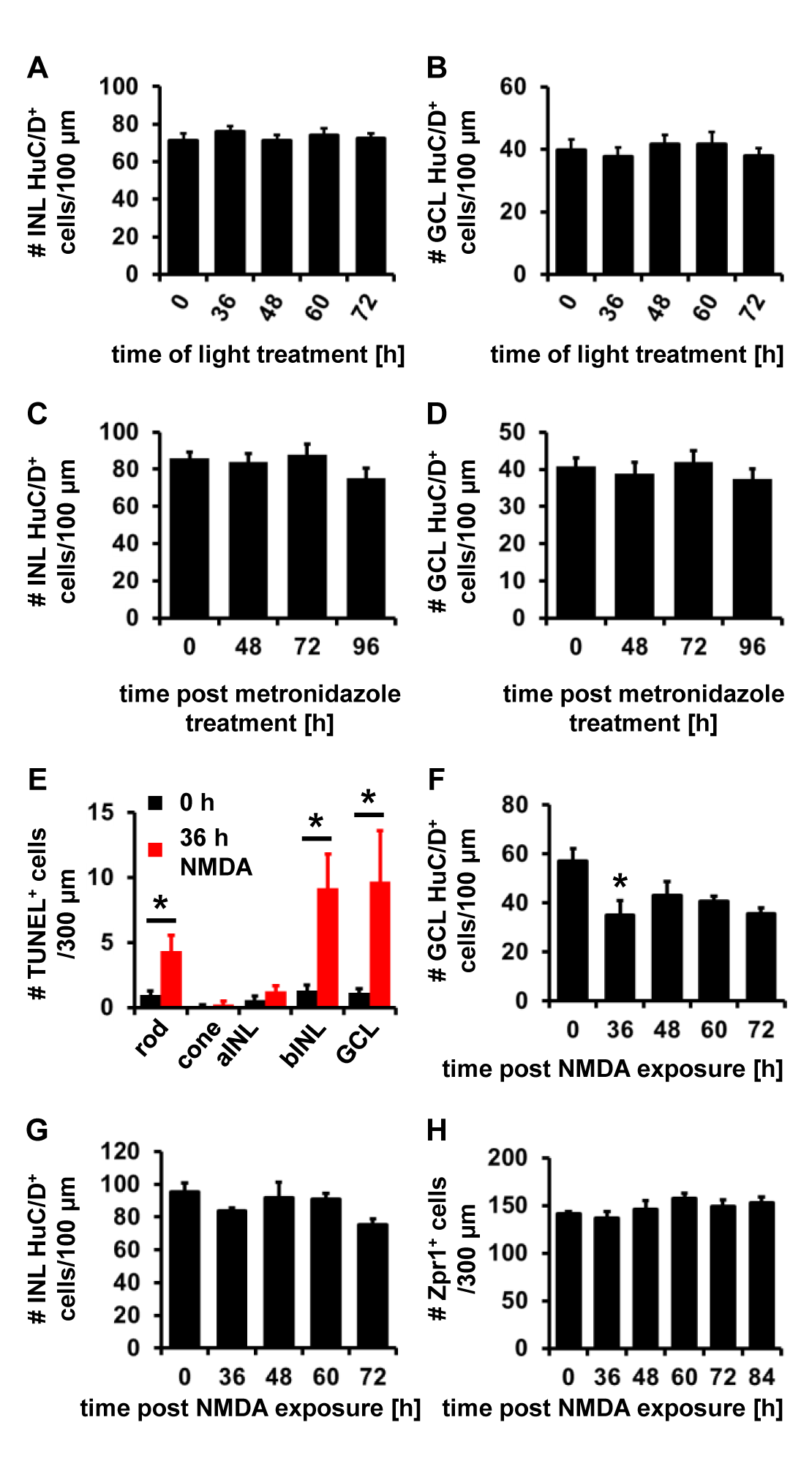

Supplement: Supplementary Figure 4 — Damage paradigms differentially affect inner retinal neuron survival. (A–D) Histograms displaying the number of HuC/D-positive cells in the INL (A,C) and GCL (B,D) following light-damage (A,B; 0, 36, 48, 60, 72 hLT) and metronidazole exposure of Tg[rho:Eco.nsfB-EGFP]nt19 zebrafish (C,D; 0, 48, 72, 96 h post mto). E) TUNEL-positive cells in the undamaged retina and at 36 h after NMDA exposure in the rod and cone nuclear layers, the apical and basal INL and the GCL. (F–H) Histograms displaying the number of HuC/D-positive cells in the GCL (F) and INL (G) and the number of Zpr-1-positive cells (H) in NMDA-damaged retinas. Mean ± SE, n > 8. pt–test < 0.05 in E, pANOVA = 0.04, pTukey < 0.05 in F. aINL, apical inner nuclear layer, bINL, basal inner nuclear layer; GCL, ganglion cell layer. [file Image_4.tiff]
