# Supplementary material for: The Regenerating Adult Zebrafish Retina Recapitulates Developmental Fate Specification Programs
Source: Front Cell Dev Biol. 2021 Feb 1;8:617923. doi: 10.3389/fcell.2020.617923 (PMC7882614; doi:10.3389/fcell.2020.617923)
Supplement: Supplementary file 5 [file Table_1.DOCX]

| **Table 1** | | |
| --- | --- | --- |
| **Gene** | **Forward primer** | **Reverse primer** |
| *18S* | 5′-CGGCTACCACATCCAAGGAAGGCAGC-3′ | 5′-TTGCTGGAATTACCGCGGCTGCT  GGCA -3′ |
| *alcama* | 5’-TCCCGAGTAAGAACCTCACCG-3’ | 5’-TTTCCTGGTTTGGGCTTGTCCT-3’ |
| *atoh7* | 5’-ACATCATGGCCCTCAATCGG-3’ | 5’-AAGCGTGCAGTCACTTTCCA-3’ |
| *barhl2* | 5’-ACGGCATCAGGCCCAAACTA-3’ | 5’-AACGGACTGGTGGACTGTCT-3’ |
| *bhlhe22* | 5’-GCTCATGACGGACGGAAGAA-3’ | 5’-TCCCGGGCGTTGATGTTTAG-3’ |
| *dlx2a* | 5’-ATCTCTGGGCCTCACGCAAA-3’ | 5’-AGACTCACTGGAGGCCACAT-3’ |
| *elavl3* | 5’-GAAGACAGGACAGGCTCTGC-3’ | 5’-GAATCTGAAGCGCTGGGTCT-3’ |
| *nrl* | 5’-CTATGCACAGCCACTCAGTCC-3’ | 5’-CAGCTGCTCGTCGGAGAAAC-3’ |
| *otx2* | 5’-GGCATCGGCTTGAATCCAGT-3’ | 5’-GCTGCTTCGGTCTCTTTTCC-3’ |
| *pcna* | 5’-TACTCAGTGTCTGCTGTGGTTTCC-3’ | 5’-CATTTAATAAGTGCGCCCGC-3’ |
| *prdm1a* | 5’-CTCTATGTGTGGCTGGGACC-3’ | 5’-ATTGTCAGCGGTGTAGGGTG-3’ |
| *ptf1a* | 5’-CCCACACAGTGACGCCTTA-3’ | 5’-TGAAAGAGAGTGTCCTGCGA-3’ |
| *rho* | 5’-GCTGAGCGCCACATCCA-3’ | 5’-AGGCACGTAGAATGCCGG-3’ |
| *thrb* | 5’-GGGTCATTTCAGGCCACGTA-3’ | 5’-TCGCTGACTTCATGGGCAAT-3’ |
| *vsx1* | 5’-CGTGTTTTCTCCCGAGCCA-3’ | 5’-ACCGGAAAGGCAGTCATCAT-3’ |
