# Supplementary material for: The Regenerating Adult Zebrafish Retina Recapitulates Developmental Fate Specification Programs
Source: Front Cell Dev Biol. 2021 Feb 1;8:617923. doi: 10.3389/fcell.2020.617923 (PMC7882614; doi:10.3389/fcell.2020.617923)
Supplement: Supplementary file 6 [file Table_2.DOCX]

**Table 2: Quantification of transgene co-localization with PCNA, HuC/D or both in *Tg[atoh7:GFP]^rw021^* and *Tg[ptf1a:EGFP]^jh1^* in light-damaged zebrafish retinas. Data are displayed as mean ± S.E. (n-number).**

| **Region/**    **Time**  **Experiment** | **INL** | | | | **ONL** | | | | **GCL** | | | |
| --- | --- | --- | --- | --- | --- | --- | --- | --- | --- | --- | --- | --- |
|  | **60 hLT** | **72**  **hLT** | **84 hLT** | **96 hLT** | **60 hLT** | **72 hLT** | **84 hLT** | **96 hLT** | **60 hLT** | **72 hLT** | **84 hLT** | **96 hLT** |
| **# *atoh7:GFP*^+^ & PCNA^+^** | 41.99 ± 6.39 (12) | 54.32 ± 5.34 (15) | 52.17 ± 6.83 (15) | 27.41 ± 4.80 (13) | 16.66 ± 5.79 (12) | 33.91 ± 4.77 (15) | 51.18 ± 7.75 (15) | 30.81 ± 6.15 (13) | 0.55 ± 0.27 (12) | 6.11 ± 2.57 (15) | 5.22 ± 1.01 (15) | 6.43 ± 1.88 (13) |
| **# HuC/D^+^ & PCNA^+^ *(Tg[atoh7:GFP])*** |  | 4.73 ± 1.11 (15) | 16.43 ± 3.76  (15) | 11.42 ± 2.13 (13) |  |  |  |  |  | 1.66 ± 0.73 (15) | 4.87 ± 0.92 (15) | 3.10 ± 1.21 (13) |
| **# *atoh7:GFP*^+^, PCNA^+^ & HuC/D^+^** |  | 4.26 ± 1.01 (15) | 16.15 ± 3.60 (15) | 8.34 ± 1.86 (13) |  |  |  |  |  | 1.59 ± 0.68 (15) | 4.52 ± 0.77 (15) | 2.83 ± 1.18 (13) |
| **# *ptf1a:EGFP*^+^ & PCNA^+^** | 6.12 ± 1.35 (13) | 29.17 ± 3.91 (13) | 47.12 ± 5.15 (10) | 26.25 ± 5.54 (11) | 1.82 ± 0.58 (13) | 23.12 ± 4.20 (13) | 30.18 ± 5.83 (10) | 7.38 ± 1.51 (11) | 0 ± 0 (13) | 0.27 ± 0.12 (13) | 1.07 ± 0.56 (10) | 0.29 ± 0.12 (11) |
