# Supplementary material for: The Regenerating Adult Zebrafish Retina Recapitulates Developmental Fate Specification Programs
Source: Front Cell Dev Biol. 2021 Feb 1;8:617923. doi: 10.3389/fcell.2020.617923 (PMC7882614; doi:10.3389/fcell.2020.617923)
Supplement: Supplementary file 7 [file Table_3.DOCX]

**Table 3:** ANOVA p-values corresponding to the data presented in the graphs.

|  |  | **ONL** | **INL** | **GCL** |
| --- | --- | --- | --- | --- |
| Light damage | *atoh7:GFP* | 1.1*10^-16^ | 1.1*10^-16^ | 3.7*10^-11^ |
|  | *atoh7:GFP* & PCNA | 2.15*10^-14^ | 1.1*10^-16^ | 5.3*10^-5^ |
|  | *atoh7:GFP* & HuC/D | 8.0*10^-9^ | 1.1*10^-16^ | 7.3*10^-15^ |
|  | PCNA & HuC/D | 0.2096 | 1.7*10^-11^ | 6.9*10^-8^ |
|  | *ptf1a:EGFP* | 1.1*10^-16^ | 1.1*10^-16^ | 6.2*10^-5^ |
|  | *ptf1a:EGFP* & PCNA | 3.7*10^-15^ | 1.1*10^-16^ | 0.0019 |
|  | *ptf1a:EGFP* & HuC/D | 1.1*10^-9^ | 5.2*10^-12^ | 7.9*10^-9^ |
|  | PCNA & HuC/D | 3.0*10^-8^ | 1.1*10^-16^ | 3.9*10^-13^ |
|  | *thrb:Tomato* | 1.1*10^-16^ | 0.0008 | not determined |
|  | *thrb:Tomato* & PCNA | 1.1*10^-16^ | 0.0006 | not determined |
|  | Zpr-1 (rod ONL) | 5.1*10^-7^ | not determined | not determined |
|  | *thrb:Tomato* & Zpr-1 & PCNA | 0.0007 | not determined | not determined |
|  | *vsx1:GFP* | 4.8*10^-11^ | not determined | 5.7*10^-9^ |
|  | *vsx1:GFP* & PCNA | 1.6*10^-11^ | 1.1*10^-16^ | 6.5*10^-7^ |
|  | TUNEL | 1.1*10^-16^ | aINL: 0.003  bINL: 0.043 | 0.11 |
| NMDA damage | *atoh7:GFP* | not determined | 6.1*10^-9^ | not determined |
|  | *atoh7:GFP* & PCNA | not determined | 2*10^-11^ | 1.7*10^-7^ |
|  | *atoh7:GFP* & HuC/D | not determined | 2.3*10^-12^ | 6.9*10^-6^ |
|  | PCNA & HuC/D | not determined | 3*10^-12^ | 3.1*10^-5^ |
|  | *ptf1a:EGFP* | not determined | 1.7*10^-13^ | not determined |
|  | *ptf1a:EGFP* & PCNA | not determined | 3.9*10^-9^ | 0.12 |
|  | *ptf1a:EGFP* & HuC/D | not determined | 2.2*10^-6^ | 0.3830 |
|  | PCNA & HuC/D | not determined | 2*10^-8^ | 0.0004 |
|  | *thrb:Tomato* (rod ONL) | 3.1*10^-5^ | not determined | not determined |
|  | *thrb:Tomato* & PCNA (rod ONL) | 0.0016 | not determined | not determined |
|  | Zpr-1 (rod ONL) | 9.3*10^-7^ | not determined | not determined |
|  | *thrb:Tomato* & Zpr-1 & PCNA (rod ONL) | 0.0094 | not determined | not determined |
