# Supplementary material for: The Regenerating Adult Zebrafish Retina Recapitulates Developmental Fate Specification Programs
Source: Front Cell Dev Biol. 2021 Feb 1;8:617923. doi: 10.3389/fcell.2020.617923 (PMC7882614; doi:10.3389/fcell.2020.617923)
Supplement: Supplementary file 8 [file Table_4.DOCX]

**Table 4:** p-values for the overall and pairwise Likelihood Ratio Tests for comparing transgene expression in PCNA-positive cells over time.

|  | | **Light damage** | | **NMDA** | |
| --- | --- | --- | --- | --- | --- |
|  |  | **# transgene^+^ & PCNA^+^** | **# transgene^+^ PCNA^+^/PCNA^+^** | **# transgene^+^ & PCNA^+^** | **# transgene^+^ PCNA^+^/PCNA^+^** |
| **p-value** | **Likelihood Ratio Test (overall)** | < 0.0001 | < 0.0001 | < 0.0001 | < 0.0001 |
|  | ***atoh7:GFP* v. *ptf1a:EGFP*** | < 0.0001 | < 0.0001 | < 0.0001 | < 0.0001 |
|  | ***atoh7:GFP* v. *thrb:Tomato*** | < 0.0001 | < 0.0001 | < 0.0001 | < 0.0001 |
|  | ***atoh7:GFP* v. *vsx1:GFP*** | < 0.0001 | < 0.0001 | < 0.0001 | < 0.0001 |
|  | ***ptf1a:EGFP* v. *thrb:Tomato*** | 1 | 1 | < 0.0001 | < 0.0001 |
|  | ***ptf1a:EGFP* v. *vsx1:GFP*** | < 0.0001 | < 0.0001 | < 0.0001 | < 0.0001 |
|  | ***thrb:Tomato* v. *vsx1:GFP*** | < 0.0001 | < 0.0001 | 0.76 | < 0.0001 |
