# Supplementary material for: The Regenerating Adult Zebrafish Retina Recapitulates Developmental Fate Specification Programs
Source: Front Cell Dev Biol. 2021 Feb 1;8:617923. doi: 10.3389/fcell.2020.617923 (PMC7882614; doi:10.3389/fcell.2020.617923)
Supplement: Supplementary file 9 [file Table_5.DOCX]

**Table 5:** Time to 10% peak expression and the corresponding lower and upper limits of the 95% confidence intervals analyzed for the number of transgene-expressing PCNA-positive cells following light or NMDA damage.

|  | | **Time to 10% peak expression [h]** | **S.E.*1.96** | **95% confidence interval** | |
| --- | --- | --- | --- | --- | --- |
|  |  |  |  | **lower** | **upper** |
| **Light damage** | ***atoh7:GFP*** | 46.78 | 3.4 | 43.38 | 50.18 |
|  | ***ptf1a:EGFP*** | 59.23 | 1.89 | 57.34 | 61.12 |
|  | ***thrb:Tomato*** | 59.84 | 2.28 | 57.56 | 62.12 |
|  | ***vsx1:GFP*** | 69.65 | 1.77 | 67.88 | 71.42 |
| **NMDA** | ***atoh7:GFP*** | 56.45 | 6.22 | 50.23 | 62.67 |
|  | ***ptf1a:EGFP*** | 58.58 | 5.43 | 53.15 | 64.01 |
|  | ***thrb:Tomato*** | 70.59 | 4.12 | 66.47 | 74.71 |
|  | ***vsx1:GFP*** | 73.27 | 5.98 | 67.29 | 79.25 |
