# Supplementary material for: The Regenerating Adult Zebrafish Retina Recapitulates Developmental Fate Specification Programs
Source: Front Cell Dev Biol. 2021 Feb 1;8:617923. doi: 10.3389/fcell.2020.617923 (PMC7882614; doi:10.3389/fcell.2020.617923)
Supplement: Supplementary file 10 [file Table_6.DOCX]

**Supplementary Table 1:** Time to 10% peak expression and the corresponding lower and upper limits of the 95% confidence intervals analyzed for the number of transgene-expressing PCNA-positive cells normalized to the number of PCNA-positive cells following light or NMDA damage.

|  | | **Time to 10% peak expression [h]** | **S.E.*1.96** | **95% confidence interval** | |
| --- | --- | --- | --- | --- | --- |
|  |  |  |  | **lower** | **upper** |
| **Light damage** | ***atoh7:GFP*** | 41.29 | 4.02 | 37.27 | 45.31 |
|  | ***ptf1a:EGFP*** | 55.1 | 2 | 53.1 | 57.1 |
|  | ***thrb:Tomato*** | 58.2 | 3.45 | 54.75 | 61.65 |
|  | ***vsx1:GFP*** | 69.3 | 1.99 | 67.31 | 71.29 |
| **NMDA** | ***atoh7:GFP*** | 50.34 | 6.28 | 44.06 | 56.62 |
|  | ***ptf1a:EGFP*** | 57.89 | 4.09 | 53.8 | 61.98 |
|  | ***thrb:Tomato*** | 68.5 | 3.53 | 64.97 | 72.03 |
|  | ***vsx1:GFP*** | 63.51 | 72.96 | -9.45 | 136.47 |
